# Intraindividual Variability of Event-Related Potentials in Psychosis: A Registered Report

**DOI:** 10.1016/j.bpsgos.2024.100396

**Published:** 2024-09-21

**Authors:** Amanda Holbrook, Bohyun Park, Philippe Rast, Gregory A. Light, Peter E. Clayson

**Affiliations:** a Department of Psychology, University of South Florida, Tampa, Florida; b Department of Psychology, University of California Davis, Davis, California; c VISN 22 Mental Illness Research, Education, & Clinical Center (MIRECC), San Diego VA Healthcare System, San Diego, California; d Department of Psychiatry, University of California San Diego, San Diego, California

**Keywords:** ERP psychometric reliability, Event-related potentials (ERPs), Individual differences, Psychiatric symptoms, Psychosis, Within-person variability

## Abstract

**Background:**

Neurophysiological tools have yielded valuable insights into the pathophysiology and treatment of psychosis. However, studies using event-related potentials (ERPs) have primarily focused on mean scores and neglected the within-person variability of ERP scores. The neglect of within-person variability of ERPs in the search for biomarkers might have resulted in crucial differences related to psychosis being missed. In this registered report, we aimed to determine whether distinct patterns of intraindividual variability in ERP biomarkers would be observed in people with a lifetime psychosis diagnosis.

**Methods:**

Publicly available data posted to the National Institute of Mental Health Data Archive for 1R01MH110434-01 was obtained for 162 patients with a lifetime history of psychosis and 178 never-psychotic (NP) participants. Participants completed tasks that measured the auditory mismatch negativity (MMN), P300, error-related negativity, and reward positivity. Multilevel location-scale models were used to determine whether patients showed greater intraindividual variability of ERP scores than NP participants.

**Results:**

Contrary to predictions, the groups did not differ in within-person variability of MMN frequency, P300, or error-related negativity; patients showed less variability in MMN duration than NP participants. Exploratory analyses of a subset of patients with schizophrenia showed greater variability of MMN in this group than in the NP group. Greater severity of thought disorder and activation symptoms were associated with higher intraindividual MMN variability.

**Conclusions:**

Distinct patterns of intraindividual variability in the measured ERPs were not observed for the broad group of people with lifetime psychotic disorders. Exploratory analyses suggest that intraindividual differences in ERPs are more relevant to schizophrenia and certain symptom dimensions than to psychotic disorders broadly, but research is needed to confirm these exploratory findings.

## Plain Language Summary

Brain responses are typically examined to understand features of psychotic disorders. Traditionally, such research has focused on the average response and has overlooked how these responses vary within an individual. In this study, we examined variability in brain responses using data from 162 people with psychosis and 178 people without psychosis to identify unique patterns of variability related to group. Contrary to expectations, variability in most brain responses was similar across groups, although some variations in response to auditory stimuli were observed in a subgroup of patients with schizophrenia. These findings suggest that variability in brain responses to auditory information could be specific to subtypes of psychosis, such as schizophrenia.

Neurophysiological tools are critical for identifying biomarkers and enhancing our understanding and treatment of neuropsychiatric disorders. For example, event-related potential (ERP) biomarkers of auditory information processing, such as the mismatch negativity (MMN) and P300, are sensitive to and predictive of treatment outcomes following cognitive training in schizophrenia (1,2). However, most ERP studies of psychosis have relied solely on average ERP scores between conditions, persons, or groups, while treating neural variability as noise. This approach overlooks the potential significance of neural variability, which may reflect the capacity to dynamically adjust from moment to moment. For example, two participants could have the same average ERP score, but one could show consistent ERP scores across trials while the other exhibits high variability. Neglecting within-person variability (i.e., intraindividual variability) in the search for biomarkers could lead to missed insights into individual differences related to schizophrenia or its treatment prognosis. Theoretical explanations of schizophrenia emphasize instability of information processing [e.g., (3, 4, 5)], which may be reflected in ERP amplitudes. This registered report describes prespecified analyses designed to determine whether distinct patterns of intraindividual variability in a battery of ERP biomarkers would be observed in people with psychosis as a prelude to further evaluating the potential of intraindividual variability of ERP scores as predictive therapeutic biomarkers.

Clinical differences in neural variability may reflect disruptions in key mechanisms relevant to dysfunction in psychopathology (6,7). For example, individuals with autism spectrum disorders show excessive neural variability despite normal mean responses, implying that such variability may contribute to unstable perception of social and emotional information (8, 9, 10). Meta-analytic work on ERP psychometrics indicates that people with psychopathology typically require more data to obtain adequate internal consistency (11), possibly due to greater intraindividual variability of ERPs. In a functional magnetic resonance imaging study, patients with schizophrenia generally showed greater neural variability than healthy control (HC) participants (12). Similarly, ERP studies of schizophrenia have shown that variability in manifestations of auditory midbrain activity distinguished patients from HC participants (13), and this variability was related to measures of speech discriminability in the context of cognitive interventions (14), thereby providing support for studies of excessive neural variability in psychosis. These findings suggest that neural variability is an important dimension in the future use of ERP biomarkers in neuropsychiatric disorders.

While several ERP components have been extensively characterized in people with or at high clinical risk for psychosis [e.g., (15,16)], intraindividual variability has rarely been assessed. Meta-analytic work indicates that people with psychosis generally show reduced duration-deviant MMN (MMN-D) (17, 18, 19), frequency-deviant MMN (MMN-F) (17, 18, 19), auditory P300 (20,21), and error-related negativity (ERN) (22); reward positivity (RewP) amplitude seems normal, but this meta-analysis was based on only 5 studies (22).

Studies of MMN and P300 provide evidence suggesting higher variability in ERP responses in psychosis. For example, auditory MMN shows greater intraindividual variability in patients with schizophrenia than in HC participants independent of mean score differences, possibly indicative of unstable auditory perception (23, 24, 25). Similarly, studies of P300 have shown high intraindividual variability of P300 in people with schizophrenia compared with HC participants (26, 27, 28) and people with depression (29). Higher P300 variability has been shown to be related to higher negative symptoms and lower cognitive function in schizophrenia, suggesting that variability could offer insights into the dynamic modulation of neural activity (26).

Despite evidence of higher intraindividual variability in MMN and P300 in schizophrenia, studies of ERN and RewP have neglected intraindividual variability as a signal of interest. We sought to determine whether instability of information processing in psychosis is indexed by ERPs related to auditory processing, performance monitoring, and reward processing. This registered report used publicly available data to test whether intraindividual variability in ERP scores corresponds to clinical group differences (psychosis vs. never psychotic). Based on findings of excessive variability of MMN and P300 in schizophrenia [e.g., (23)] and meta-analytic work on the clinical group differences (17, 18, 19, 20, 21, 22), we predicted that patients with a lifetime history of psychosis would show higher intraindividual variability than the never-psychotic (NP) comparison group in the following ERPs: MMN (MMN-D and MMN-F), P300 (P3a and P3b), and ERN, but not RewP^1^.

## Methods and Materials

The current article is a registered report. As part of this registration, we adhered to recommendations for open science practices (30,31). All study materials for data processing and analysis are posted to Open Science Framework (https://osf.io/qyjs5/). Analyses were conducted on publicly available data posted to the National Institute of Mental Health Data Archive (https://nda.nih.gov/edit_collection.html?id=2477) for the “Trajectories of Aging in Psychotic Disorders Over 27 Years” National Institute of Mental Health grant awarded to Roman Kotov, Ph.D. (1R01MH110434-01).

### Participants

Participants were recruited from psychiatric facilities in Suffolk County, New York, between 1989 and 1995 as part of the Suffolk County Mental Health Project (32). Eligibility criteria at baseline included the presence of psychosis and an age range of 15 to 60 years. Data in the current analyses were collected at the 20-year follow-up. At the 20-year follow-up, age- and gender-matched NP participants without a lifetime history of psychosis or psychiatric hospitalization were recruited. The initial sample size for data obtained from the National Institute of Mental Health Data Archive was 569 participants. Inclusion criteria for the current analyses included the following: 1) diagnostic information verified by clinical interview (see the Clinical Assessments section), 2) clinical symptom ratings (see the Clinical Assessments section), and 3) at least 5 trials for each ERP condition (see the Data Inclusion section).

### Clinical Assessments

Psychiatric diagnoses were assessed using the Structured Clinical Interview for DSM-IV Axis I disorders-Patient Edition (33), which was administered by master’s-level interviewers. Symptom levels were assessed using the Scale for the Assessment of Positive Symptoms (34), the Scale for the Assessment of Negative Symptoms (35), and the Brief Psychiatric Rating Scale (36). Scoring for each measure is described in the Clinical Assessments section of the Supplement.

### Experimental Tasks

Established paradigms, which are described in detail in the Supplement, were used to elicit each ERP. An auditory MMN paradigm was used to elicit MMN-D and MMN-F (37), an auditory oddball task was used to elicit P300 (38), a modified version of the Eriksen flanker task was used to record ERN (39), and a doors task was used for measuring RewP (40).

### Electrophysiological Data Recording and Reduction

Procedures for recording and reducing data are described in full in the Supplement. Continuous electroencephalography was recorded from 34 active scalp electrodes placed based on the 10/20 system using an ActiveTwo BioSemi amplifier (BioSemi). Electroencephalography was filtered online using a low-pass filter of 204.8 Hz. Electrooculogram was recorded from additional sensors placed above and below the eyes and near the outer canthi.

Data were algebraically re-referenced offline to averaged mastoids and then filtered with half-amplitude cutoffs at 0.01 and 30 Hz. Stimulus-locked or response-locked epochs were extracted. Ocular artifacts were removed using independent component analysis, and bad channels were identified and interpolated. The first 200 ms of epochs were used for baseline adjustment.

Studies of ERPs in psychosis, including published studies from this dataset, guided the selection of time windows and sensors for scoring ERN (41,42), RewP (42,43), P300 (44,45), and MMN (37,46).

#### Mismatch Negativity

Stimulus-locked epochs were extracted from 200 ms before the onset of the standard and deviant tones to 500 ms after the onset of tones. A collapsed localizer approach was used to identify the time windows for scoring MMN (see the Supplement). Time-window mean amplitude windows from 265 to 315 ms at Fz were used for scoring MMN-D, and mean amplitude windows from 175 to 225 ms were used for scoring MMN-F.

#### P300

Stimulus-locked epochs for novel sounds and correct response to target tones were extracted from 200 ms before sounds/tones to 800 ms following tones/sounds. For P3a, single-trial time-window mean amplitudes were extracted from 250 to 450 ms at Cz following novel sounds; for P3b, single-trial time-window mean amplitudes were extracted from 300 to 500 ms at Pz following target tones.

#### Error-Related Negativity

Response-locked epochs for correct and error trials were extracted from 400 ms prior to participant response to 800 ms following participant response. Single-trial ERP scores were extracted using a time-window mean amplitude from 0 to 100 ms at FCz.

#### Reward Positivity

Stimulus-locked epochs for gain and loss trials were extracted from 200 ms before feedback presentation to 800 ms after feedback presentation. Single-trial ERP scores were extracted using a time-window mean amplitude from 250 to 350 ms at FCz.

### Data Inclusion

To ensure that enough data were retained per person for robust variance estimates, only participants with at least 5 trials per ERP condition were included in each ERP analysis step. This 5-trial threshold for inclusion was chosen based on analyses of the robustness of the multilevel location-scale models (see the Multilevel Models section in the Supplement) that indicate high statistical power, good coverage, and minimal-to-zero bias with at least 10 observations per person for *n* = 250 [see documentation for (47)]. Another simulation analysis of multilevel location-scale models indicates that the models yield sufficient power (>0.80) with only 10 total observations per person for *n* = 200 and for very small effect sizes (48). Therefore, if a minimum of 5 trials were retained per ERP condition for a person (i.e., 10 total trials), there should be sufficient data for robust variance estimates.

### Data Analysis

Estimates of group- and participant-level psychometric internal consistency and data quality (standardized measurement error) are reported to characterize observed ERP data (49). Generalizability theory equations were used to estimate coefficients of dependability [see (50, 51, 52, 53, 54, 55)]. Dependability as a function of the number of trials needed for a stable average ERP score was estimated separately for each event type, difference score, group, and ERP using the ERP Reliability Analysis Toolbox (56,57). Arithmetically derived estimates of the standard error of the mean were used to characterize the data quality of each ERP score (58).

Bayesian multilevel models were used to examine single-trial ERP scores (59, 60, 61). These models account for the unbalanced nature of ERP data due to trials being excluded because of artifact rejection or unbalanced events (e.g., correct vs. error trials) by partially pooling information across parameters to improve their estimation (62,63). By partially pooling information across participants, extreme observations (e.g., possibly those with very few trials) were shrunk closer to the group mean because participants from the same population are expected to be similar to each other.

The location-scale generalization of multilevel models (48) was used to simultaneously estimate means and residual variances of ERP scores. Bayesian location-scale models have been successfully applied in studies of ERPs (53,64, 65, 66, 67, 68, 69) and have been advocated as tools to better understand intraindividual variability of ERPs (70)^2^. Briefly, location-only multilevel models estimate individual mean scores but hold the residual variance constant. The location-scale generalization expands the multilevel structure and includes a model for the residual variance (i.e., scale), allowing differences in intraindividual variability. This permits the modeling of fixed and random effects for residual variance. The Bayesian models and their priors are described in the Supplement. Each model for each ERP included predictors for event type, diagnostic group, and the interaction. The location-scale models were used to determine whether clinical group differences accounted for intraindividual variability of ERP scores.

### Manipulation Check

To justify analysis and interpretation of intraindividual variability of ERP scores, location-scale models were expected to show improved model fit over location-only models. Leave-one-out cross-validation via Pareto smoothed importance sampling was used to compare model fits (71) using the R package *loo* (72). Conceptually speaking, leave-one-out cross-validation via Pareto smoothed importance sampling compares the predictive accuracy of models. A given model is expected to show better fit over another model when 1) the difference in expected log predictive density (${\hat{elpd}}_{loo}$) is >4, and 2) the difference in ${\hat{elpd}}_{loo}$ is >2 times the standard error of ${\hat{elpd}}_{loo}$ (SE(${\hat{elpd}}_{loo}$)). Two models are considered to have comparable fits when 1) the difference in ${\hat{elpd}}_{loo}$ is <4, or 2) the difference in ${\hat{elpd}}_{loo}$ is <2 times the SE(${\hat{elpd}}_{loo}$).

### Primary Test of Hypothesis

We predicted that patients with psychosis would show higher intraindividual variability than the comparison group in the following ERPs: MMN, P300 (P3a and P3b), and ERN, but not RewP.

The hypotheses for MMN, P300 (P3a and P3b), and ERN would be supported if 1) the 95% credible interval (CrI) for the group parameter on the scale portion of the model does not contain zero, and the psychosis group shows higher variability; and/or 2) the 95% CrI for the Event × Group parameter on the scale portion of the model does not contain zero, the psychosis group shows higher variability for one event, and the groups show comparable variability for the other event.

The hypothesis for RewP would be supported if 1) the 95% CrI for the group parameter on the scale portion of the model contains zero, and 2) the 95% CrI for the Event × Group parameter on the scale portion of the model contains zero.

### Exploratory Analyses

Location-scale models were used to examine the relationship between intraindividual variability of ERP scores and psychiatric symptoms in patients and HC participants. These models included predictors for event type, psychiatric symptoms, and the Event × Symptom interaction (see the Supplement). Models also predict post-error slowing from previous-trial ERN amplitudes (see the Supplement).

### Deviations From Preregistration

The preregistration erroneously stated that only patients with missing symptom-level data would be excluded from analysis, but this exclusion criterion was applied to all participants.

## Results

A total of 340 participants (162 patients, 178 NP participants) had usable electroencephalography data for at least 1 task and were included in analyses. See Table 1 for demographic and diagnostic characteristics of the sample and Figure S1 for a description of data attrition. Groups were similar in gender distribution (χ^2^_1_ = 0.11, *p* = .74). Groups differed in age (*t*_338_ = −4.08, *p* < .001) such that patients were 4 years younger on average than NP participants. Groups differed in racial distribution (χ^2^_4_ = 13.924, *p* = .01) such that the patient group included a higher proportion of racial minorities than the NP group. The patient group included a broad range of psychotic disorders (Table 1).

**Table 1:** Demographic, Diagnostic, BPRS, and SAPS/SANS Summary Statistics by Group

Measure	Patients, *n* = 162		NP Participants, *n* = 178	
Age, Years	52.9 (8.59)		56.8 (8.93)	
Sex				
Female	73 (45.06%)		84 (47.19%)	
Male	89 (54.94%)		94 (52.81%)	
Race				
Asian	4 (2.47%)		0 (0.00%)	
Black/African American	21 (12.96%)		10 (5.62%)	
White	136 (83.95%)		162 (91.01%)	
More Than One Race	0 (0.00%)		2 (1.12%)	
Unknown	1 (0.62%)		4 (2.25%)	
Diagnosis	Lifetime	Past-Month	Lifetime	Past-Month
Schizophrenia	58 (35.80%)	57 (35.19%)	0 (0.00%)	0 (0.00%)
Schizoaffective disorder	22 (13.58%)	21 (12.96)	0 (0.00%)	0 (0.00%)
Bipolar I	51 (31.48%)	7 (4.32%)	0 (0.00%)	0 (0.00%)
Bipolar II	1 (0.62%)	0 (0.00%)	1 (0.56%)	1 (0.56%)
Other bipolar^a^	5 (3.09%)	1 (0.62%)	0 (0.00%)	0 (0.00%)
Delusional disorder	1 (0.62%)	1 (0.62%)	0 (0.00%)	0 (0.00%)
Substance-induced psychotic disorder	7 (4.32%)	0 (0.00%)	0 (0.00%)	0 (0.00%)
Psychotic disorder NOS	7 (4.32%)	0 (0.00%)	0 (0.00%)	0 (0.00%)
Major depressive disorder^b^	15 (9.26%)^c^	5 (3.09%)	37 (20.79%)^d^	6 (3.37%)
Depressive disorder NOS^e^	7 (4.32%)	1 (0.62%)	6 (3.37%)	1 (0.56%)
Substance-induced mood disorder	1 (0.62%)	0 (0.00%)	0 (0.00%)	0 (0.00%)
Dysthymic disorder	2 (1.23%)	2 (1.23%)	5 (2.81%)	5 (2.81%)
BPRS				
Affect	8.02 (4.03)		6.62 (3.06)	
Positive symptoms	7.23 (4.52)		4.28 (0.81)	
Negative symptoms	6.49 (3.09)		4.6 (1.27)	
Resistance	5.52 (3.09)		3.73 (1.5)	
Activation	4.24 (1.77)		3.43 (0.97)	
SAPS/SANS				
Thought disorder	5.58 (5.93)		1.71 (2.66)	
Reality distortion	3.38 (5.2)		0.05 (0.32)	
Apathy/asociality	13.1 (7.67)		4.73 (5.9)	
Inexpressivity	7.77 (8.92)		1.82 (3.4)	

Values are presented as mean (SD) or *n* (%).

BPRS, Brief Psychiatric Rating Scale; NOS, not otherwise specified; NP, never-psychotic; SANS, Scale for the Assessment of Negative Symptoms; SAPS, Scale for the Assessment of Positive Symptoms.

a Three of 5 patients with other bipolar diagnoses had lifetime psychotic disorders.

b Four participants with lifetime major depressive disorder had lifetime psychotic disorders.

c Among patients with major depressive disorder, 3 experienced a single episode, 8 experienced recurrent episodes, and 4 were unknown.

d For NP participants with major depressive disorder, 15 experienced a single episode, 15 experienced recurrent episodes, and 7 were unknown.

e All 7 participants with lifetime depressive disorder NOS had lifetime psychotic disorders. It was not clear from the National Data Archive that the additional 11 participants with major depressive disorder, 2 with dysthymic disorder, 1 with substance-induced mood disorder, 1 with bipolar II disorder, and 2 with other bipolar disorder had psychotic features; however, it can be assumed given that all patients at baseline were hospitalized for psychosis.

Grand average waveforms are presented in Figures S2 to S4. Summary information, internal consistency, and data quality estimates for ERP scores are reported in Tables S1, S6, and S7.

### Manipulation Check

Each location-scale model showed improved model fit over its corresponding location-only model based on the log predictive density (${\hat{elpd}}_{loo})$, justifying the interpretation of within-subject variability using location-scale models. See Table S2 for a summary of model fits.

### Model Interpretation

#### Duration-Deviant Mismatch Negativity

Parameter estimates for the MMN-D model are presented in Table 2, and pairwise contrasts are provided in Table 3. MMN-D was larger (i.e., more negative) on deviant trials than on standard trials for patients (95% CrI: −2.43 to −1.89) and NP participants (95% CrI: −2.87 to −2.37) (see Figure S2). Patients exhibited smaller (i.e., less negative) MMN-D for deviant trials (95% CrI: 0.03 to 0.92) and ΔMMN-D (95% CrI: 0.10 to 0.82) than NP participants. Groups did not differ in amplitude for standards corresponding to the MMN-D time window. For the scale portion of the model, MMN-D deviants were approximately 1% more variable than standards for NP participants (95% CrI: 0.004 to 0.02). Patients showed 2% less variability in ΔMMN-D than NP participants (95% CrI: −0.03 to −0.004), the opposite of the pattern that was predicted.

**Table 2:** Estimates From Location-Scale Multilevel Model Predicting MMN-D and MMN-F Amplitude

Parameter	Estimate	SE	95% CrI
MMN-D			

Location Portion			
Standard: NP participants (intercept)	1.63	0.11	1.42 to 1.84
Standard: patients	0.02	0.16	−0.28 to 0.33
Deviant: NP participants	−2.61	0.13	−2.87 to −2.37
Deviant: patients	0.46	0.19	0.10 to 0.82
Scale Portion (SD)			
Standard: NP participants (intercept)	2.49	0.02	2.45 to 2.53
Standard: patients	0.04	0.03	−0.02 to 0.10
Deviant: NP participants	0.01	0.004	0.01 to 0.02
Deviant: patients	−0.02	0.01	−0.03 to −0.004
Random Effects (SD)			
Mean structure			
Standard (intercept)	1.34	0.07	1.22 to 1.48
Deviant	1.39	0.08	1.24 to 1.54
Variance structure			
Standard (intercept)	0.27	0.01	0.25 to 0.29
Deviant	0.02	0.01	0.01 to 0.03

MMN-F			

Location Portion			
Standard: NP participants (intercept)	−0.44	0.12	−0.68 to −0.19
Standard: patients	0.44	0.17	0.11 to 0.78
Deviant: NP participants	−3.36	0.13	−3.61 to −3.11
Deviant: patients	0.57	0.18	0.21 to 0.93
Scale Portion (SD)			
Standard: NP participants (intercept)	2.43	0.02	2.39 to 2.47
Standard: patients	0.04	0.03	−0.02 to 0.09
Deviant: NP participants	0.01	0.01	−0.003 to 0.02
Deviant: patients	−0.01	0.01	−0.03 to 0.002
Random Effects (SD)			
Mean structure			
Standard (intercept)	1.53	0.07	1.40 to 1.68
Deviant	1.41	0.08	1.27 to 1.57
Variance structure			
Standard (intercept)	0.26	0.01	0.24 to 0.28
Deviant	0.04	0.004	0.03 to 0.05

Estimates of parameters represent the median, and parameters in standard deviation (SD) units are shown on a log scale. The intercept for the location portion of the model represents mean ERP score for NP participants for the reference level (standard trials). Subsequent estimates represent the deviation from the intercept. For example, for MMN-D, the point estimate for standard: patients (0.02) indicates that the mean ERP score for patients for standard trials is 0.02 μV from the intercept. The intercept for the scale portion represents average within-person variability for the NP group, and subsequent estimates for the scale portion represent change from the intercept on the log scale. Positive coefficients on the scale portion of the model indicate greater variability, and negative coefficients represent less variability. Random effects for the location portion of the model represent person-specific departures of means around the grand mean (mean structure), and random effects for the scale portion of the model represent person-specific departures of variability around the grand mean variability (variance structure).

CrI, credible interval; ERP, event-related potential; MMN-D, duration-deviant mismatch negativity; MMN-F, frequency-deviant MMN; NP, never-psychotic.

**Table 3:** Pairwise Contrasts for Group-Related Differences

Parameter	Median	95% CrI	Parameter	Median	95% CrI
Location Portion of Models					
MMN-D			MMN-F		
Standard	0.02	(−0.28 to 0.33)	Standard	0.43^a^	(0.11 to 0.78)^a^
Deviant	0.48^a^	(0.03 to 0.92)^a^	Deviant	1.00^a^	(0.50 to 1.51)^a^
Deviant-Standard	0.46^a^	(0.10 to 0.82)^a^	Deviant-Standard	0.56^a^	(0.21 to 0.93)^a^
P3a			P3b		
Target	0.74	(−0.06 to 1.56)	Target	0.48	(−0.29 to 1.25)
Novel	0.07	(−0.83 to 0.97)	Nontarget	−0.31	(−0.71 to 0.08)
Novel-Target	−0.67	(−1.40 to 0.03)	Target-Nontarget	0.80^a^	(0.10 to 1.50)^a^
RewP			ERN		
Loss	0.98	(−0.44 to 2.39)	Correct	−2.99^a^	(−4.38 to −1.62)^a^
Gain	0.41	(−1.26 to 2.07)	Error	−0.85	(−2.16 to 0.48)
Loss-Gain	−0.57	(−1.55 to 0.44)	Error-Correct	2.15^a^	(1.02 to 3.26)^a^

Scale Portion of Models					
MMN-D			MMN-F		
Standard	0.04	(−0.02 to 0.10)	Standard	0.02	(−0.04 to 0.09)
Deviant	0.02	(−0.04 to 0.08)	Deviant	0.04	(−0.02 to 0.09)
Deviant-Standard	−0.02^a^	(−0.03 to −0.004)^a^	Deviant-standard	0.01	(−0.002 to 0.03)
P3a			P3b		
Target	−0.01	(−0.07 to 0.06)	Target	−0.01	(−0.08 to 0.06)
Novel	−0.04	(−0.10 to 0.02)	Nontarget	0.002	(−0.06 to 0.06)
Novel-Target	−0.03	(−0.09 to 0.03)	Target-Nontarget	−0.02	(−0.07 to 0.03)
RewP			ERN		
Loss	0.003	(−0.07 to 0.08)	Correct	−0.01	(−0.11 to 0.09)
Gain	−0.01	(−0.08 to 0.07)	Error	−0.01	(−0.11 to 0.08)
Loss-Gain	−0.01	(−0.07 to 0.06)	Error-Correct	−0.002	(−0.05 to 0.05)

All estimates are for the patient minus NP group contrasts.

CrI, credible interval; ERN, error-related negativity; MMN-D, duration-deviant mismatch negativity; MMN-F, frequency-deviant MMN; NP, never-psychotic; RewP, reward positivity.

a Denotes the 95% CrI of the contrast excludes zero. This is interpreted as a difference.

#### Frequency-Deviant Mismatch Negativity

Parameter estimates for the MMN-F model are presented in Table 2, and pairwise contrasts are provided in Table 3. For the location portion of the model, MMN-F scores were larger (i.e., more negative) on deviant trials than on standard trials for patients (95% CrI: −3.07 to −2.52) and NP participants (95% CrI: −3.61 to −3.11) (Figure S3). Patients exhibited smaller (i.e., less negative) MMN-F deviant (95% CrI: 0.50 to 1.51), MMN-F standard (95% CrI: 0.11 to 0.78), and ΔMMN-F (95% CrI: 0.21 to 0.93) than NP participants. For the scale portion of the model, none of the covariates predicted variability in MMN-F amplitudes, and therefore, our hypothesis was not supported.

#### P3a

Parameter estimates for the P3a model are presented in Table 4, and pairwise contrasts are provided in Table 3. For the location portion of the model, P3a was larger on novel trials than target trials for patients (95% CrI: 0.93 to 1.98) and NP participants (95% CrI: 1.62 to 2.64), but group differences were not observed (Figure S3). For the scale portion of the model, none of the predictors predicted within-person variability, thus failing to support our hypothesis for P3a.

**Table 4:** Estimates From Location-Scale Multilevel Model Predicting P3a and P3b Amplitude

Parameter	Estimate	SE	95% CrI
P3a			

Location Portion			
Target: NP participants (intercept)	1.31	0.29	0.75 to 1.87
Target: patient	0.75	0.42	−0.06 to 1.56
Novel: NP participants	2.13	0.26	1.62 to 2.64
Novel: patients	−0.67	0.36	−1.40 to 0.03
Scale Portion (SD)			
Target: NP participants (intercept)	2.35	0.02	2.30 to 2.39
Target: patients	−0.01	0.03	−0.07 to 0.06
Novel: NP participants	0.01	0.02	−0.03 to 0.05
Novel: patients	−0.03	0.03	−0.09 to 0.03
Random Effects (SD)			
Mean structure			
Target (intercept)	3.01	0.18	2.67 to 3.38
Novel	1.28	0.32	0.62 to 1.88
Variance structure			
Target (intercept)	0.25	0.01	0.22 to 0.27
Novel	0.14	0.02	0.10 to 0.18

P3b			

Location Portion			
Target: NP participants (intercept)	2.3	0.27	1.77 to 2.84
Target: patients	0.48	0.39	−0.29 to 1.25
Nontarget: NP participants	−0.92	0.25	−1.41 to −0.44
Nontarget: patients	−0.79	0.36	−1.50 to −0.10
Scale Portion (SD)			
Target: NP participants (intercept)	2.33	0.02	2.28 to 2.38
Target: patients	−0.01	0.04	−0.08 to 0.06
Nontarget: NP participants	−0.03	0.02	−0.06 to 0.01
Nontarget: patients	0.02	0.03	−0.03 to 0.07
Random Effects (SD)			
Mean structure			
Target (intercept)	2.8	0.18	2.47 to 3.17
Nontarget	2.29	0.18	1.95 to 2.65
Variance structure			
Target (intercept)	0.28	0.01	0.25 to 0.31
Nontarget	0.16	0.01	0.13 to 0.18

Estimates of parameters represent the median, and parameters in standard deviation units are shown on a log scale. The intercept for the location portion of the model represents the mean ERP score for NP participants for the reference level (target trials). Subsequent estimates represent the deviation from the intercept. For example, for P3a, the point estimate for target: patients (0.75) indicates that the mean ERP score for patients for standard trials is 0.75 μV from the intercept. The intercept for the scale portion represents average within-person variability for the NP group, and subsequent estimates for the scale portion represent change from the intercept on the log scale. Positive coefficients on the scale portion of the model indicate greater variability, and negative coefficients represent less variability than NP participants. Random effects for the location portion of the model represent person-specific departures of means around the grand mean (mean structure), and random effects for the scale portion of the model represent person-specific departures of variability around the grand mean variability (variance structure).

CrI, credible interval; ERP, event-related potential; NP, never-psychotic.

Analyses were repeated using P3a scored using a collapsed localizer approach, and the pattern of effects was similar. An additional group difference in amplitude for the location portion of the model was found such that patients showed smaller novel and difference P3a than NP participants (see the Supplement).

#### P3b

Parameter estimates for the P3b model are presented in Table 4, and pairwise contrasts are provided in Table 3. P3b was larger for target than for nontarget trials for patients (95% CrI: −2.25 to −1.19) and NP participants (95% CrI: −1.41 to −0.44) (Figure S3). Patients showed smaller P3b difference scores than NP participants (95% CrI: −1.50 to −0.10), but P3b differences between groups were not observed to constituent events. For the scale portion of the model, none of the predictors predicted within-person variability, thus failing to support our hypothesis for P3b.

Analyses were repeated using P3b scored using a collapsed localizer approach. Results were similar except that group differences were not found for the location portion of the model. Additionally, greater within-person variability was found for P3b target scores than for nontarget scores in patients and NP participants (see the Supplement).

#### Error-Related Negativity

Parameter estimates for the ERN model are presented in Table 5, and pairwise contrasts are provided in Table 3. For the location portion of the model, both patients (95% CrI: −2.53 to −0.89) and NP participants (95% CrI: −4.64 to −3.02) exhibited larger (i.e., more negative) ERN on error trials than on correct trials (Figure S4). Patients showed larger ERN on correct trials (95% CrI: −4.38 to −1.62) than NP participants and smaller (i.e., less negative) ΔERN (95% CrI: 1.02 to 3.26) than NP participants. Error-trial ERN differences were not observed between groups. For the scale portion of the model, the within-person variability of ERN scores for error and correct trials was similar. Group differences in the within-person variability of ERN scores were not observed, thus failing to support our hypothesis for ERN.

**Table 5:** Estimates From Location-Scale Multilevel Model Predicting ERN and RewP Amplitude

Parameter	Estimate	SE	95% CrI
ERN			

Location Portion			
Correct: NP participants (intercept)	5.99	0.48	5.05 to 6.92
Correct: patients	−2.99	0.7	−4.38 to −1.62
Error: NP participants	−3.85	0.41	−4.64 to −3.02
Error: patients	2.14	0.57	1.02 to 3.26
Scale Portion (SD)			
Correct: NP participants (intercept)	2.43	0.03	2.36 to 2.50
Correct: patients	−0.01	0.05	−0.11 to 0.09
Error: NP participants	0.02	0.02	−0.02 to 0.05
Error: patients	−0.002	0.02	−0.05 to 0.05
Random Effects (SD)			
Mean structure			
Correct (intercept)	5.94	0.26	5.45 to 6.48
Error	4.35	0.25	3.88 to 4.87
Variance structure			
Correct (intercept)	0.42	0.02	0.39 to 0.46
Error	0.14	0.02	0.11 to 0.17

RewP			

Location Portion			
Loss: NP participants (intercept)	8.08	0.49	7.12 to 9.03
Loss: patients	0.98	0.72	−0.44 to 2.39
Gain: NP participants	2.91	0.35	2.23 to 3.58
Gain: patients	−0.57	0.51	−1.55 to 0.44
Scale Portion (SD)			
Loss: NP participants (intercept)	2.32	0.03	2.27 to 2.37
Loss: patients	0.003	0.04	−0.07 to 0.08
Gain: NP participants	0.03	0.02	−0.02 to 0.07
Gain: patients	−0.01	0.03	−0.07 to 0.06
Random Effects (SD)			
Mean structure			
Loss (intercept)	5.75	0.29	5.20 to 6.36
Gain	2.45	0.31	1.84 to 3.06
Variance structure			
Loss (intercept)	0.26	0.02	0.23 to 0.29
Gain	0.09	0.03	0.02 to 0.15

Estimates of parameters represent the median, and parameters in standard deviation units are shown on a log scale. The intercept for the location portion of the model represents the mean ERP score for NP participants for the reference level (correct or loss trials). Subsequent estimates represent the deviation from the intercept. For example, for ERN, the point estimate for correct: patients (−2.99) indicates that the mean ERP score for patients for standard trials is 2.99 μV from the intercept. The intercept for the scale portion represents average within-person variability for the NP group, and subsequent estimates for the scale portion represent change from the intercept on the log scale. Positive coefficients on the scale portion of the model indicate greater variability, and negative coefficients represent less variability than NP participants. Random effects for the location portion of the model represent person-specific departures of means around the grand mean (mean structure), and random effects for the scale portion of the model represent person-specific departures of variability around the grand mean variability (variance structure).

CrI, credible interval; ERN, error-related negativity; ERP, event-related potential; NP, never-psychotic; RewP, reward positivity.

#### Reward Positivity

Parameter estimates for the RewP model are presented in Table 5, and pairwise contrasts can be found in Table 3. For the location portion of the model, RewP gain was larger than RewP loss for patients (95% CrI: 1.56 to 3.10) and NP participants (95% CrI: 2.23 to 3.58) (Figure S4). Amplitude differences between groups were not identified for RewP gain, RewP loss, or ΔRewP scores. None of the predictors on the scale portion of the model predicted within-person variability, which supported our hypothesis for RewP.

### Exploratory Analyses

A full summary of exploratory analyses looking at the relationship between the within-subject variability of ERP components and symptoms measured with the Brief Psychiatric Rating Scale and Scale for the Assessment of Positive Symptoms/Scale for the Assessment of Negative Symptoms is presented in the Supplement (see the Supplement for beta coefficients). Briefly, higher levels of thought disorder symptoms were related to greater within-subject variability of RewP on loss trials but not gain trials. Higher activation symptoms were related to greater within-person variability for MMN-D and MMN-F on standard trials but not deviant trials. Higher levels of thought disorder symptoms were related to greater within-person variability for MMN-F scores on both standard trials and deviant trials.

Exploratory analyses were conducted to examine group differences between patients with schizophrenia and HC participants. Patients showed 11% more variability for MMN-D for standard trials (95% CrI: 0.01 to 0.22) and 11% more variability for MMN-F for deviant trials (95% CrI: 0.01 to 0.21) than HC participants.

## Discussion

In this registered report, we aimed to describe the intraindividual variability of commonly used ERP components in broad psychotic disorders, expanding on the extensive literature that primarily focuses on mean ERP component amplitudes. Despite observing the expected attenuation in mean ERN, P3, and MMN in the patient group, these differences were not accompanied by consistent alterations in within-person variability. Contrary to our predictions, the groups did not differ in within-person variability of ERN, P3, or MMN-F. An opposite pattern from the one that was predicted was found for MMN-D; patients showed less variability than NP participants. Consistent with predictions, RewP scores were similar in patients and NP participants. Therefore, intraindividual variability of ERN, P3, and MMN did not consistently differentiate people with lifetime psychotic disorders from NP comparison participants.

Although higher intraindividual variability of MMN (23) and P300 (26, 27, 28) has been observed in schizophrenia, the current findings suggest that these effects do not extend to patients with diverse psychotic disorders, including affective and nonaffective psychotic disorders. High intraindividual variability of MMN and P300 may be specific to patients with schizophrenia rather than psychosis broadly, as supported by exploratory analyses for MMN in a subset of patients with schizophrenia (see the Supplement). Another reason for the discrepancy with other research could be the use of location-scale multilevel models; other studies have compared standard error of the mean (23) or standard deviations (26). Multilevel location-scale models simultaneously model the mean response (location) and the residual variability (scale) while controlling for each other’s presence and accounting for correlations among mean and variance, a feature that is lost when comparing sample standard errors of the mean and standard deviations.

Symptom-level analyses suggest that increased intraindividual variability of ERP component amplitudes was related to specific symptom dimensions of psychosis. For example, higher thought disorder composite scores from the Scale for the Assessment of Positive Symptoms/Scale for the Assessment of Negative Symptoms were related to greater variability of RewP and MMN-F, and higher activation symptoms on the Brief Psychiatric Rating Scale were related to greater variability of MMN-D and MMN-F. These findings indicate that specific symptom dimensions may contribute to higher intraindividual variability. When considering these findings together with comparable group effects for intraindividual variability, it is possible that group differences in variability did not emerge due to the lower severity of psychiatric symptoms in the current sample than in other studies [e.g., (23,27)]. However, considering the many exploratory analyses conducted, the observed ERP-symptom relationships warrant replication in independent samples.

The current findings have important methodological implications. Improved model fit of location-scale models over traditional (location only) models indicates notable intraindividual variability. This underscores the importance of accounting for intraindividual variability rather than relying solely on average ERPs because failing to account for intraindividual variability can lead to mistaken statistical inferences (48,73). In other words, ignoring this variability could obscure true differences between groups or misrepresent relationships between neural activity and clinical phenomena (70).

The following limitations of the current study should be considered. First, patients with psychotic disorders were chronically ill, making it unclear whether our findings would generalize to individuals in the early course of the illness. Notably, patients were 20 years older on average than patients from studies reporting increased intraindividual variability of MMN (23). Second, patients were likely on antipsychotic and other medications, which could have affected ERP findings, but this is speculative^3^. Our prior large-scale consortium studies of schizophrenia have shown that medication regimens are complex and affect cognition (74). In fact, higher cumulative anticholinergic medication burden across multiple classes of medications has been associated with reduced MMN and P3a (75). P300 in patients with schizophrenia is comparable to HC participants when patients were treated with antipsychotic medications (76), highlighting the need to consider medication usage. Third, the current study did not account for the presence of psychiatric diagnoses beyond psychotic and mood disorders. The presence of additional diagnoses in the patient and NP groups might have influenced the results. For example, anxiety and depression symptoms are associated with alterations in ERP components, particularly ERN and RewP (77,78). Nonetheless, the current study had notable strengths, including the use of a registered report format, analysis of a large sample for an ERP study (79,80), and the use of an open dataset that supports transparency and reproducibility.

### Conclusions

Taken together, the findings from this registered report indicated that intraindividual variability of ERP components seems more relevant to certain symptom dimensions in psychosis than to the diagnostic status of participants. The potential specificity of greater intraindividual variability of MMN may be specific to patients with schizophrenia as opposed to psychotic disorders broadly, but this suggestion should be considered speculative given the exploratory nature of the analyses. Further research is needed to confirm these observations and examine their clinical implications, emphasizing the need for a nuanced approach in the neurophysiological study of psychotic disorders and psychiatric symptoms.

## Supplementary Material

Key Resources Table

## Footnotes

1 We did not predict differences in intraindividual variability of RewP in schizophrenia due to consistent findings of similar amplitudes to HC participants. When there are consistent between-group findings in mean scores, high variability in scores would likely affect average scores for the group and lead to differences in some analyses, but this does not seem to be the case for studies of RewP.

## Footnotes

2 A discussion of location-scale models and their application to ERPs is described in the Supplement of Clayson *et al.* (64).

3 This information was not included in the online data repository.
