# Supplementary material for: Intraindividual Variability of Event-Related Potentials in Psychosis: A Registered Report
Source: Biol Psychiatry Glob Open Sci. 2024 Sep 21;5(1):100396. doi: 10.1016/j.bpsgos.2024.100396 (PMC11609361; doi:10.1016/j.bpsgos.2024.100396)
Supplement: Supplement [file mmc2.pdf]

SUPPLEMENTARY INFORMATION

Intraindividual Variability of Event-Related Potentials in Psychosis: A Registered Report

Holbrook *et al.*

Supplementary Method and Materials

Clinical Assessments

The following sections contain information about clinical assessment composite scores used for present analyses.

Brief Psychiatric Rating Scale (BPRS). Individual items on the BPRS are rated from 1 (*absent*) to 7 (*extremely severe*). Analyses used five subscales based on meta-analytic work on the factor structure of the BPRS (1). The Affect subscale comprises anxiety and depressive symptoms, somatic concerns, and feelings of guilt. The Positive Symptoms subscale comprises unusual thought content, hallucinatory behavior, grandiosity, and conceptual disorganization. The Negative Symptoms subscale comprises blunted affect, emotional withdrawal, disorientation, and motor retardation. The Resistance subscale comprises hostility, suspiciousness, and uncooperativeness. The Activation subscale comprises mannerisms and posturing, tension, and excitement.

Scale for the Assessment of Positive/Negative Symptoms (SAPS/SANS). Items on the SAPS/SANS are rated from 0 (*absent*) to 5 (*severe*). Composite scores for Thought Disorder, Reality Distortion, Apathy/Asociality, and Inexpressivity were used for analysis based on previous factor analyses (2, 3).The Inexpressivity composite comprises items from the SANS affective flattening subscale (unchanging facial expression, decreased spontaneous movement, paucity of expressive gestures, poor eye contact, affective nonresponsivity, lack of vocal inflections) and SANS alogia subscale (poverty of speech, increased latency of response)[1]. The Reality Distortion composite comprises items from the SAPS delusions subscale (grandiose delusions, religious delusions, ideas and delusions of reference, delusions of being controlled,

[1] The Inexpressivity composite score originally included a social inattentiveness item from the SANS in the original factor analysis (2). However, data for this item was unavailable in the dataset obtained from the NDA.

delusions of mind reading, thought broadcasting, thought insertion, thought withdrawal) and SAPS hallucinations subscale (auditory hallucinations, voices commenting, voices conversing, somatic or tactile hallucinations, olfactory hallucinations, visual hallucinations). The apathy/asociality composite comprises items from the SANS anhedonia/asocialty subscale (recreational interests and activities, sexual interest and activity, ability to feel intimacy and closeness, relationships with friends and peers) and SANS avolition/apathy subscale (impersistence at work or school, physical anergia). The Thought Disorder composite comprises items from the SAPS positive formal thought disorder subscale (loose associations, tangentiality, incoherence (word salad), illogicality, circumstantiality, pressure of speech, distractible speech, clanging), the SAPS bizarre behavior subscale (social and sexual behavior), and the SANS alogia subscale (inappropriate affect, poverty of content of speech).

Experimental Tasks

The following sections contain detailed information about each task used for the present analyses.

Auditory MMN. The auditory MMN task is described in a recent publication of the present dataset (4). Participants were presented with auditory stimuli while viewing a picture/word matching task. Auditory stimuli were presented with a fixed inter-stimulus interval of 500 ms at 75 dB with a 10 ms rise/fall. Of the 2,458 tones, 245 (10%) deviated in duration (100 ms at 633 Hz), and 245 (10%) deviated in frequency (50 ms at 1000 Hz).

Flanker task. A modified version of the Eriksen flanker task (5) was used, and the task of the original study is described elsewhere (6). On each trial, five horizontally aligned white arrowheads were presented, with the arrows in the array either all pointed in the same direction (i.e., congruent trial: >>>>> or <<<<<) or with the central arrow pointing in the opposite

direction of other arrows (i.e., incongruent trial: <<><< or >><>>). The arrows were presented for 200 ms and were followed by an inter-trial interval that varied randomly from 2300-2800 ms. Participants were instructed to press the left or right mouse button, corresponding to the direction of the center arrow, and respond in such a way as to maximize speed and accuracy. Participants first completed a practice block of 30 trials; the actual task consisted of 11 blocks of 330 trials. Trial blocks consisted of 50% congruent and 50% incongruent trials.

Performance-based feedback was presented at the end of each block to encourage fast and accurate behavior. If performance accuracy was below or equal to 75%, the feedback “Please try to be more accurate” was shown. If accuracy was above 90%, the feedback “Please try to respond faster” was shown. Otherwise, if accuracy was above 75 and less than or equal to 90%, the feedback “You’re doing a great job” was shown.

Doors task. Each trial begins with the presentation of two identical images of doors. Participants were instructed to select the left or right door by clicking the left or right mouse button, respectively. Participants were informed that they could win $0.50 or lose $0.25 on each trial and that they would be paid their total winnings at the end of the task. The images of the doors were presented until the participants selected a door. Then, a fixation cross was displayed for 1000 ms, which was followed by a feedback stimulus presentation for 2000 ms. The feedback stimulus was indicated by a green arrow pointing upwards following gains and a red arrow pointing downwards following losses, respectively. Then, another fixation cross was presented for 1500 ms, followed by the prompt “Click for next round”, until participants responded with a button click to initiate the next trial, ensuring that participants remained actively engaged during the task. The task consisted of three 20-trial blocks (60 trials total), with 10 gain and 10 loss trials per block.

Auditory Oddball. A three-stimulus auditory oddball task was used to elicit P3 (e.g., 7). Participants wore headphones in a sound-attenuated dark room and were instructed to count the number of times a synthesized "you" sound was presented in each block. The frequent nontarget (i.e., standard) stimulus was a synthesized "me" sound. Rare novel stimuli were chosen at random from a set of 25 non-repeating unique sounds (e.g., a flute noise) or synthesized noises (e.g., "beep"). Each stimulus was separately presented for 200 ms at 75 dB and the interval between sound onsets was 1500 ms. There were 250 trials divided into five blocks (50 trials per block). Overall, 10% of stimuli were targets, 80% were frequent nontargets, and 10% were novels. Participants first were introduced to the "you" and "me" sounds, and then completed a practice block to provide an opportunity to ask questions. Participants reported the number of targets they heard at the end of each block. Accuracy was calculated as the sum of deviations from the correct number of targets in each block and expressed as a percent.

Electrophysiological Data Recording and Reduction

Continuous EEG was recorded using an ActiveTwo BioSemi amplifier (BioSemi, Amsterdam, Netherlands). EEG signals were pre-amplified at the electrode with a gain of one and were digitized at a sampling rate of 1,024 Hz with a 24-bit analog-to-digital converter (least significant bit: 31.25 nV). EEG was filtered online using a low-pass, fifth-order sinc filter with a half-power cut-off of 204.8 Hz. EEG was recorded from 34 active scalp electrodes placed based on the 10/20 system (including FCz and Iz). Two additional scalp electrodes were placed on the left and right mastoids. Electrooculogram was recorded from additional sensors placed above and below the eyes and near the outer canthi. Electrodes were referenced online to a common mode sense electrode that formed a monopolar channel.

Data were algebraically rereferenced offline to averaged mastoids and then filtered using a sixth-order IIR Butterworth filter with half-amplitude cutoffs at .01 and 30 Hz in ERPLab 9.0.0 (8). Stimulus-locked or response-locked epochs were extracted and further information on these epochs is below. Eye blinks and horizontal and vertical saccadic eye movement were removed using independent components analysis (ICA) implemented in the ERP PCA Toolkit (9). For the ICA procedure, epoched EEG data from all channels were processed through a binary version of EEGLab's *runica* function called binica (10). Any ICA components that correlated at .8 or above with the scalp topography of a blink template or with the scalp topography of vertical and horizontal saccade templates were removed from the data. The templates used for artifact correction include those automatically generated by the ERP PCA Toolkit and those created by the present authors from the dataset.

Following artifact correction, channels with more than a 100 μV step within 100 ms intervals, a voltage difference of 300 μV through the duration of the epoch, or an absolute correlation with the nearest six neighboring channels that fell below .4 were marked as bad for the epoch. Channels marked as bad for more than 20% of epochs were considered globally bad. Bad channels will be interpolated using spherical splines (11), but if more than 10% of channels were marked bad for an epoch, the entire epoch was rejected. The first 200 ms of the stimulus- and response-locked epochs were used for baseline adjustment.

Data Analysis

MMN Scoring. Stimulus-locked epochs were extracted from 200 ms before the onset of the standard and deviant tones to 500 ms after the onset of tones. A collapsed localizer approach was used to identify the time-windows for scoring MMN because there are few studies that use the picture/word task in psychosis (12). Separate difference waveforms were created for the

duration deviant minus standard (MMN-D) and the frequency deviant minus standard (MMN-F). Grand average waveforms for MMN-D and MMN-F collapsed across group were used to identify the most negative-going peaks between 100 and 300 ms for Fz. Separate time-window mean amplitudes of 50 ms that center on the identified peaks from MMN-D and MMN-F were used to extract single-trial MMN scores for the duration deviant and standard analyses and the frequency deviant and standard analyses. The same time-windows were used for each group. Time-window mean amplitude windows from 265 to 315ms at Fz were used for scoring MMN-D and from 175 to 225 ms were used for scoring MMN-F.

P3 Scoring. The a priori scoring window for P3a (250 to 450ms) and P3b (300 to 500ms) was broad for the observed P3a, capturing a preceding negative peak, and for the observed P3b, capturing only the beginning of the positive deflection. Therefore, an exploratory analysis was used to identify time windows for scoring P3a and P3b and used a collapsed localizer approach similar to MMN (12). Grand average waveforms for P3a from Cz and for P3b from Pz collapsed across group were used to identify peaks for scoring, and the same time windows were used for both groups. Time-window mean amplitudes from 385 to 485 were used for P3a and from 425 to 525 were used for P3b.

Multilevel Models. The models used to evaluate clinical group differences and clinical symptom relationships are described here. Wilkinson notation is used for transparency (13). *Model 1* was used to evaluate clinical group differences, and *Model 2* was used to evaluate clinical symptom relationships in people with psychosis. The same parameters were fit on the location and scale portions of the model.

Model 1: Single-Trial ERP ~ 1 + Event + Group + Event x Group + (1 + Event | Participant)

Sigma ~ 1 + Event + Group + Event x Group + (1 + Event | Participant)

Model 2: Single-Trial ERP ~ 1 + Event + Symptoms + Event x Symptoms + (1 + Event | Participant)

Sigma ~ 1 + Event + Symptoms + Event x Symptoms + (1 + Event | Participant)

The Event predictor levels were duration deviant vs. standard (comparison level) for MMN-D, frequency deviant vs. standard (comparison level) for MMN-F, error vs. correct (comparison level) for ERN, gain vs. loss (comparison level) for RewP, novel sounds vs. target sounds (comparison level) for P3a, and non-target sounds vs. target sounds (comparison level) for P3b. The Group predictor was people with psychosis vs. people without psychosis (comparison level). The Symptoms predictor was either SANS, SAPS, or BPRS.

Response Times. Post-error, correct-trial response times (RTs) were predicted from ERN amplitudes to determine whether there are clinical group differences in post-error slowing and whether ERN predicts intraindividual variability in post-error slowing. Only data recorded during the flanker task was used—trial-level behavioral data was not collected during the auditory MMN or auditory oddball paradigms. Although trial-level behavioral data are collected during the doors paradigm, task performance is unrelated to whether a trial is a gain or a loss as the trial feedback was predetermined.

Bayesian models were fit within *R* (14) using the package *brms* (15), which is a front end wrapper for *Stan* (16). For ERP models, the priors for fixed effect estimates, including the

population intercepts, used a normal distribution with a mean of 0 and a standard deviation of 3. For RT models, the priors for fixed effect estimates, including the population intercepts, used a normal distribution with a mean of 500 and a standard deviation of 100. All variance components were estimated using a Student's *t* prior distribution with 10 degrees of freedom, a 0 location parameter, and a 2 scale parameter. An LKJ prior distribution with a shape parameter of 3 was used as the prior for the correlation between the varying intercepts and slopes effects of event type within persons (17). A shape = 3 parameter can be considered mildly informative, putting more weight on an identity matrix. Similar location-scale models and priors have been successfully used on models of ERN data, and they yielded good convergence and robust parameter estimates (18).

Model 3: Correct-trial RT ~ 1 + Congruency + Group + Previous-trial ERN + Congruency x Group x Previous-trial ERN + (1 + Congruency | Participant)

Sigma ~ 1 + Congruency + Group + Previous-trial ERN + Congruency x Group x Previous-trial ERN + (1 + Congruency | Participant)

The Congruency predictor included two levels: current congruent trial or current incongruent trial. The Previous-trial ERN predictor corresponds to the amplitude of ERN from the previous error trial. The three-way interaction is shown above, and the model included each lower-level two-way interactions.

Supplementary Results

Participants

Initial sample size was 569 participants. The sample unexpectedly contained three participants classified as siblings who were excluded from further analyses, resulting in 566 participants (340 patients, 226 controls). Next, participants with missing or incomplete clinical interview data (n=36) were excluded, resulting in 530 participants (312 patients, 218 controls). Of these participants, 343 had EEG data files. Participants were excluded for having unreadable EEG data files or for having too few available trials (see data inclusion) separately for doors (n=68), auditory oddball (n=28), flanker (n=58), and auditory MMN (n=29). The final task-specific participant counts were as follows: 275 for doors, 315 for auditory oddball, 285 for flanker, and 314 for auditory MMN. Data retention is presented in Supplementary Figure 1. Summary ERP data is presented by group in Supplementary Table 1 and grand average waveforms are presented in Supplementary Figures 2-4. Demographic data is presented by group for the 340 participants who had usable EEG data for at least one task in Table 1 in the main text and separately by EEG task in Supplementary Tables 2-5. Gender identity and ethnicity data were unavailable for all participants.

For symptom-level analyses, participants were excluded if more than 10% of SAPS/SANS (i.e., 4 items) or BPRS (i.e., 2 items) were missing. Any remaining missing data was replaced using mean imputation. The number of participants with data available for symptom analyses for each task was as follows: 259 for doors, 295 for auditory oddball, 269 for flanker, and 295 for auditory MMN.

Reliability

Reliability and data quality estimates for ERN, RewP, P3, MMN, post-error slowing and P3a and P3b scores using a collapsed localizer approach are presented in Supplementary Table 6 for controls and Supplementary Table 7 for patients.

Group level reliability. For RewP, patients and controls demonstrated numerically similar group-level reliability for constituent scores (ϕ = .81-.89). For ΔRewP scores, patients demonstrated numerically lower group-level reliability (ϕ = .26) than controls (ϕ = .50).

Patients showed adequate correct-trial ERN score reliability (ϕ = .97) but poor group-level error-trial (ϕ = .51) and difference score reliability (ϕ = .34). The poor reliability was primarily due to several participants with poor internal consistency that were pulling the group-level reliability estimates down. Average subject-level reliability estimates were numerically higher for error trials (ϕ = .78) than group-level estimates, because subject-level reliability estimates do not assume fixed residual variance across all participants. When residual variance is separately estimated for each participant using subject-level reliability, a few participants with high residual variance will not greatly impact the group-level residual variance estimate. For controls, group-level reliability was .79 for correct trials, .83 for error trials, and .81 for the difference score.

For P3a scores, patients showed acceptable reliability (ϕ = .76) and controls showed unacceptable reliability (ϕ = .68) for novel trials, whereas scores for neither group reached acceptable levels of reliability for target trials (<.7). P3a difference score reliability was numerically higher for patients (ϕ = .50) than controls (ϕ = .34). For P3b scores, both patients (ϕ = .84) and controls (ϕ = .83) showed excellent reliability for non-target trials, and unacceptable reliability (<.7) for target trials. P3b difference score reliability was numerically higher for

patients (ϕ = .70) than controls (ϕ = .64). P3a and P3b scored using a collapsed localizer approach demonstrated similar reliability levels as time-window mean amplitude scores for novel and non-target trials. Likewise, difference score reliability was similar across scoring approaches. The reliability of P3a and P3b for target trials scores using a collapsed localizer approach reached acceptable levels (>.7) for patients. The reliability of P3b for target trials was acceptable for controls (ϕ = .70), while the reliability of P3a for target trials was unacceptable for controls (ϕ = .67).

For MMN-D scores, controls demonstrated excellent reliability for standard (ϕ = .97) and deviant (ϕ = .89) trials, while patients displayed acceptable reliability for deviant trials (ϕ = .78) and excellent reliability for standard trials (ϕ = .93). MMN-D difference score reliability was numerically lower for patients (ϕ = .64) than controls (ϕ = .68). For MMN-F scores, excellent reliability was found for both patients and controls for standard and deviant trials (ϕ = .84-.98). MMN-F difference score reliability was numerically higher for patients (ϕ = .71) than controls (ϕ = .65).

Model 1 Fit Summary

Each location-scale model showed substantial improvement in model fit over its corresponding location-only model based on the log predictive density ($\widehat{elpd}_{loo}$). A summary of model fits for location-only and location-scale models is shown in Supplementary Table 8.

Exploratory Analyses

Group Models (Model 1)

Group analyses for P3 were repeated with P3 scored using a collapsed localizer approach. Summary information for P3a and P3b scored using this approach is presented in Supplementary Table 1.

P3a collapsed localizer. Parameter estimates for the P3a model are presented in Supplementary Table 9, and pairwise contrasts are provided in Supplementary Table 10. For the location portion of the model, P3a scores were larger (i.e., more positive) on novel than target trials for both patients (95% CrI: 0.73, 2.07) and controls (95% CrI: 2.88, 4.19). Group differences in amplitude were found such that patients had smaller P3a scores on novel trials (95% CrI: -2.39, -0.30) and smaller P3a difference scores (95% CrI: -3.05, -1.24) than controls. No group differences were found for target trials. For the scale portion of the model, neither patients nor controls showed differences between within-person variability of P3a scores for novel and target trials. No group differences in within-person variability were found for P3a scores, failing to support our hypothesis for P3a.

P3b collapsed localizer. Parameter estimates for the P3b model are presented in Supplementary Table 11, and pairwise contrasts are provided in Supplementary Table 10. For the location portion of the model, P3b scores were larger (i.e., more positive) for target than non-target trials for patients (95% CrI: -5.03, -3.68) and controls (95% CrI: -4.60, -3.35). No group differences were identified for P3b scores for target or non-target trials or their difference. For the scale portion of the model, greater within-person variability was found for P3b target scores than non-target scores for patients (95% CrI: -0.09, -0.02) and controls (95% CrI: -0.10, -0.03). No group differences in within-person variability were found for P3b scores, failing to support our hypothesis for P3b.

Symptom Models (Model 2)

MMN-D. For the location portion of the model, a main effect of symptom was found for the BPRS Affect model such that higher symptom levels were related to larger (i.e., more negative) MMN-D scores regardless of event type (95% CrI: -0.09, -0.004) (see Supplementary

Table 12). Follow-up simple slope analysis revealed BPRS Affect symptoms were related to larger MMN-D scores on standard trials (*b* = -0.05, 95% CrI: -0.09, -0.004), however, there was no relationship between BPRS Affect symptoms and MMN-D scores on deviant trials (*b* = -0.06, 95% CrI: -0.13, 0.002).

An Event x Symptom interaction was found for the following SAPS/SANS models: Apathy/Asociality (95% CrI: 0.03, 0.07), Thought Disorder (95% CrI: 0.004, 0.09), and Inexpressivity (95% CrI: 0.002, 0.06). Simple slope analyses revealed that higher apathy/ asociality (*b* = 0.05, 95% CrI: 0.02, 0.08), inexpressivity (*b* = 0.04, 95% CrI: 0.01, 0.08), and thought disorder symptoms (*b* = 0.05, 95% CrI: 0.002, 0.10) were related to smaller (i.e., less negative) MMN-D scores on deviant trials, while these symptom scores were unrelated to MMN-D scores on standard trials.

For the scale portion of the model, an Event x Symptom interaction was found for the BPRS Activation model (95% CrI: -0.01, -0.001). Simple slope analyses revealed higher BPRS Activation symptoms were related to greater within-person variability for MMN-D scores on standard trials (*b* = 0.02, 95% CrI: 0.002, 0.05) and were unrelated to within-person variability for MMN-D scores on deviant trials (*b* = 0.02, 95% CrI: -0.003, 0.04). An Event x Symptom interaction was also found for SAPS/SANS Thought Disorder model (95% CrI: -0.003, -0.001), however, follow-up simple slope analyses showed no relationship between symptoms and within-person variability of MMN-D standard (*b* = 0.01, 95% CrI: -0.00002, 0.01) or deviant scores (*b* = 0.005, 95% CrI: -0.002, 0.01).

MMN-F. For the location portion of the model, a main effect of symptom was found for the BPRS Negative Symptoms (95% CrI: 0.02, 0.17) and SAPS/SANS Inexpressivity (95% CrI: 0.02, 0.07) models such that higher symptom levels were related to smaller (i.e., less negative)

MMN-F scores regardless of event type (see Supplementary Table 13). Follow-up simple slope analyses revealed greater BPRS Negative Symptoms were significantly related to smaller MMN-F scores on standard trials (b = 0.09, 95% CrI: 0.02, 0.17), however, the relationship between negative symptoms and MMN-F scores on deviant trials (b = 0.1, 95% CrI: -0.01, 0.22) was insignificant. For the SAPS/SANS Inexpressivity model, higher symptoms were related to smaller MMN-F scores on both standard (b = 0.04, 95% CrI: 0.02, 0.07) and deviant trials (b = 0.05, 95% CrI: 0.01, 0.09).

An Event x Symptom interaction was found for the BPRS Activation (95% CrI: 0.01, 0.28) and SAPS/SANS Apathy/Asociality (95% CrI: 0.01, 0.06) and SAPS/SANS Thought Disorder (95% CrI: 0.003, 0.08) models. For BPRS Activation, follow-up simple slope analyses revealed higher symptoms were related to smaller MMN-F scores on deviant trials (b = 0.23, 95% CrI: 0.02, 0.42), while symptoms were unrelated to MMN-F scores on standard trials (b = 0.08, 95% CrI: -0.05, 0.21). For SAPS/SANS Apathy/Asociality, higher symptoms were related to smaller MMN-F scores on deviant (b = 0.07, 95% CrI: 0.03, 0.10) and standard (b = 0.03, 95% CrI: 0.01, 0.05) trials. For SAPS/SANS Thought Disorder, higher symptoms were related to smaller MMN-F scores on deviant trials (b = 0.07, 95% CrI: 0.02, 0.13), while symptoms were unrelated to MMN-F scores on standard trials (b = 0.03, 95% CrI: -0.01, 0.07).

For the scale portion of the model, a main effect of symptom was found for BPRS Activation (95% CrI: 0.002, 0.05) such that higher symptoms were related to greater within-person variability for MMN-F scores regardless of event type. Follow-up simple slope analyses revealed higher symptoms were related to greater within-person variability for MMN-F scores on standard trials (b = 0.02, 95% CrI: 0.002, 0.05) but not MMN-F scores on deviant trials (b = 0.02, 95% CrI: -0.002, 0.04). A main effect of symptom was also found for SAPS/SANS

Thought Disorder model (95% CrI: 0.0004, 0.01) such that higher symptoms were related to greater within-person variability for MMN-F scores regardless of event type. Follow-up simple slope analysis revealed higher symptoms were related to greater within-person variability for MMN-F scores on both standard (*b* = 0.01, 95% CrI: 0.0004, 0.01) and deviant trials (*b* = 0.01, 95% CrI: 0.0003, 0.01).

An Event x Symptom interaction was found for BPRS Affect model (95% CrI: -0.004, -0.0002), however, follow-up simple slopes analyses did not yield relationships between symptoms and MMN-F scores on deviant (*b* = -0.01, 95% CrI: -0.01, 0.004) and standard trials (*b* = -0.003, 95% CrI: -0.01, 0.01).

P3a. For the location portion of the model, a main effect of symptom was found for the BPRS Affect model (95% CrI: 0.001, 0.23) such that higher symptom levels were related to larger (i.e., more positive) P3a scores regardless of event type (see Supplementary Table 14). Follow-up simple slope analysis revealed higher BPRS Affect symptoms were related to larger P3a scores on target trials (*b* = 0.12, 95% CrI: 0.001, 0.23), however, there was no relationship between BPRS Affect symptoms and P3a scores on novel trials (*b* = 0.05, 95% CrI: -0.08, 0.18).

For the scale portion of the model, an Event x Symptom interaction was found for the BPRS Activation model (95% CrI: -0.05, -0.003). However, follow-up simple slope analysis failed to show relationships between BPRS Activation symptoms and P3a scores for target (*b* = 0.01, 95% CrI: -0.01, 0.04) and novel trials (*b* = -0.01, 95% CrI: -0.03, 0.01).

P3a Collapsed Localizer. For the location portion of the model, an Event x Symptom interaction was found for the BPRS Activation (95% CrI: -0.79, -0.08) model (see Supplementary Table 15). Follow-up simple slope analysis revealed higher BPRS Activation symptoms were related to greater P3a scores on target trials (*b* = 0.40, 95% CrI: 0.03, 0.77), but

not novel trials (*b* = -0.03, 95% CrI: -0.42, 0.35). An Event x Symptom interaction was also found for BPRS Affect symptoms (95% CrI: -0.29, -0.01). Follow-up simple slope analysis revealed higher BPRS Affect symptoms were significantly related to greater P3a scores on target trials (*b* = 0.16, 95% CrI: 0.01, 0.31), but not novel trials (*b* = 0.01, 95% CrI: -0.14, 0.16).

For the scale portion of the model, within-subject variability of P3a scores was not related to BPRS or SAPS/SANS subscale scores (see Supplementary Table 15).

P3b. For the location portion of the model, an Event x Symptom interaction was found for the BPRS Activation (95% CrI: -0.57, -0.05) model (see Supplementary Table 16). Follow-up simple slope analyses revealed that higher BPRS Activation symptoms were related to larger P3b scores on target trials (*b* = 0.34, 95% CrI: 0.06, 0.62), while symptoms were unrelated to P3b scores on non-target trials (*b* = 0.04, 95% CrI: -0.11, 0.18).

For the scale portion of the model, within-subject variability of P3b scores was not related to BPRS or SAPS/SANS subscale scores (see Supplementary Table 16).

P3b Collapsed Localizer. For the location portion of the model, P3b scores were not related to BPRS or SAPS/SANS subscale scores. For the scale portion of the model, within-subject variability of P3b scores was not related to BPRS or SAPS/SANS subscale scores (see Supplementary Table 17).

ERN. For the location portion of the model, a main effect of symptom was found for BPRS Resistance (95% CrI: -0.67, -0.10) (see Supplementary Table 18). Follow-up simple slope analysis revealed higher symptoms were related to larger ERN scores on correct trials (*b* = -0.39, 95% CrI: -0.67, -0.10), while symptoms were not related to ERN scores on error trials (*b* = -0.23, 95% CrI: -0.50, 0.03). A main effect of symptom was also found for SAPS/SANS Reality Distortion (95% CrI: -0.41, -0.03) model such that higher symptoms were related to larger (i.e.,

more negative) ERN scores regardless of event type. Follow-up simple slope analysis revealed higher symptoms were related to larger ERN scores on correct trials (*b* = -.22, 95% CrI: -0.41, -0.03), while symptoms were not related to ERN scores on error trials (*b* = -.07, 95% CrI: -0.23, 0.10).

An Event x Symptom interaction was found for the BPRS Negative Symptoms (95% CrI: 0.06, 0.50) and the following SAPS/SANS models: Inexpressivity (95% CrI: 0.07, 0.24), Apathy/Asociality (95% CrI: 0.09, 0.24), and Thought Disorder (95% CrI: 0.07, 0.33). Follow-up simple slope analyses revealed that higher levels of BPRS Negative Symptoms (*b* = -.45, 95% CrI: -0.72, -0.17), SAPS/SANS Inexpressivity (*b* = -.19, 95% CrI: -0.28, -0.09), SAPS/SANS Apathy/asociality (*b* = -.17, 95% CrI: -0.27, -0.08), and SAPS/SANS Thought Disorder symptoms (*b* = -.19, 95% CrI: -0.34, -0.02), were each related to larger (i.e., more negative) ERN scores on correct trials, while symptoms were not related to ERN scores on error trials.

For the scale portion of the model, within-subject variability of ERN scores was not related to BPRS or SAPS/SANS subscale scores (see Supplementary Table 18).

RewP. For the location portion of the model, a main effect of symptom was found for the BPRS Affect model (95% CrI: 0.03, 0.43) such that higher symptom levels were related to larger (i.e., more positive) RewP scores regardless of event type (see Supplementary Table 19). Follow-up simple slope analysis revealed higher BPRS Affect symptoms were related to larger RewP scores on loss trials (*b* = .23, 95% CrI: 0.03, 0.43), however, there was no relationship between BPRS Affect symptoms and RewP scores on gain trials (*b* = .19, 95% CrI: -0.04, 0.43).

For the scale portion of the model, a main effect of symptom was found for the SAPS/SANS Thought Disorder model (95% CrI: 0.0001, 0.02) such that higher symptom levels were related to greater within-person variability of RewP scores regardless of event type.

Follow-up simple slope analysis revealed higher SAPS/SANS Thought Disorder scores were related to greater within-subject variability of RewP scores on loss trials ($b = 0.01$, 95% CrI: 0.0001, 0.02), however, there was no relationship between SAPS/SANS Thought Disorder symptoms and RewP scores on gain trials ($b = 0.01$, 95% CrI: -0.002, 0.01).

Post-Error Slowing (Model 3)

For the location portion of the model, neither patients nor controls showed differences between RTs on congruent and incongruent trials (i.e., congruency effect; see Supplementary Table 16). For group comparisons, contrasts of posterior samples were used to compare congruent, incongruent, and difference score conditions between controls and patients. Patients demonstrated slower RTs on congruent (95% CrI: 19.43, 78.54) and incongruent trials (95% CrI: 14.61, 79.21) than controls (see Supplementary Table 20). Patients and controls did not differ on incongruent minus congruent RTs. To examine the relationship between previous-trial ERN and trial congruency, a simple slope analysis was performed. Neither group showed a relationship between ERN and RTs for congruent or incongruent trials or their difference. Moreover, simple slope contrasts revealed no group differences.

For the scale portion of the model, neither patients nor controls showed differences in within-person variability for RT on congruent versus incongruent trials. For group comparisons, contrasts of posterior samples were used to compare congruent, incongruent, and difference score conditions between patients and controls. Patients showed 24% greater within-person variability of RTs on congruent trials (95% CrI: 0.09, 0.37) and 27% greater within-person variability of RTs on incongruent (95% CrI: 0.14, 0.40) than controls. Patients and controls did not differ on incongruent minus congruent within-person variability. To examine the relationship between previous-trial ERN and congruency, a simple slope analysis was performed. Although

patients showed greater variability in the ERN difference score contrast (95% CrI: 0.0004, 0.01), neither patients nor controls showed that ERN difference scores predicted post-error slowing within group (i.e., simple slopes analyses did not yield within-group differences despite indicating between-group differences).

Schizophrenia-only Model (Model 1)

Group-level Model 1 analyses were performed on patients with only schizophrenia (n=58) and healthy controls without any diagnosis (n=129)[2]. Pairwise contrasts are presented in Supplementary Table 21. Similar to the full sample, patients with schizophrenia showed a larger ERN for correct trials and a smaller ΔERN than controls. Likewise, patients demonstrated smaller MMN-D for deviant trials, ΔMMN-D, MMN-F for standard and deviant trials, and ΔMMN-F scores than controls. Contrary to the full sample, patients with schizophrenia did not show a smaller P3b difference score than controls.

Patients showed 11% more variability for MMN-D for standard trials (95% CrI: 0.01, 0.22) and 11% more variability for MMN-F for deviant trials (95% CrI: 0.01, 0.21) than controls did. Parameter estimates for the MMN-D and MMN-F models are presented in Supplementary Tables 22 and 23 respectively.

---

[2] Based only on diagnoses for which data was available (see Table 1 in main text). The number of participants included for each task is as follows: Doors: 43 patients, 118 controls; Flanker: 45 patients, 117 controls; Auditory Oddball: 53 patients, 129 controls; Auditory MMN: 52 patients, 76 controls.

Supplementary Figure 1

Data Retention Summary

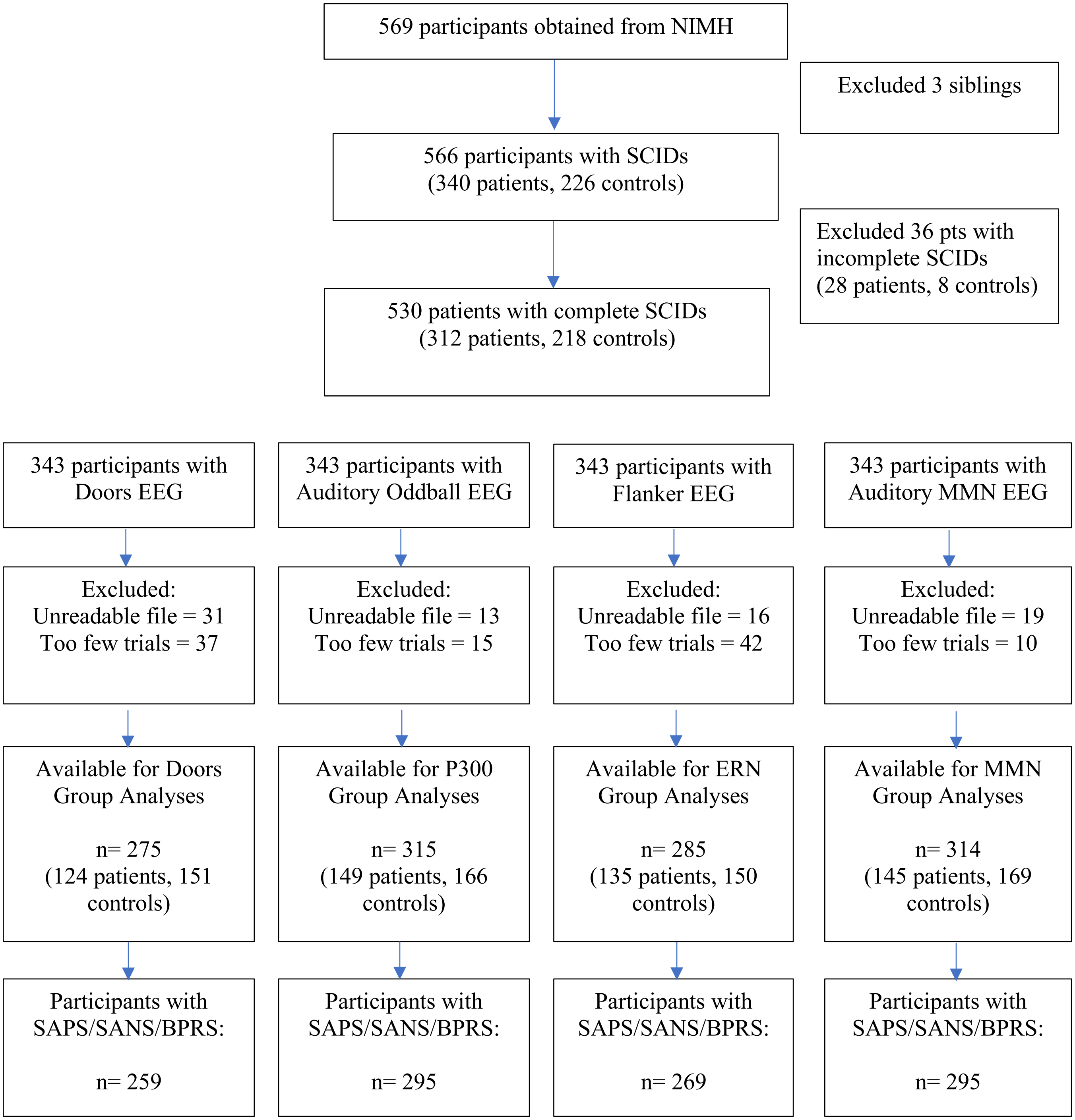

Supplementary Figure 2

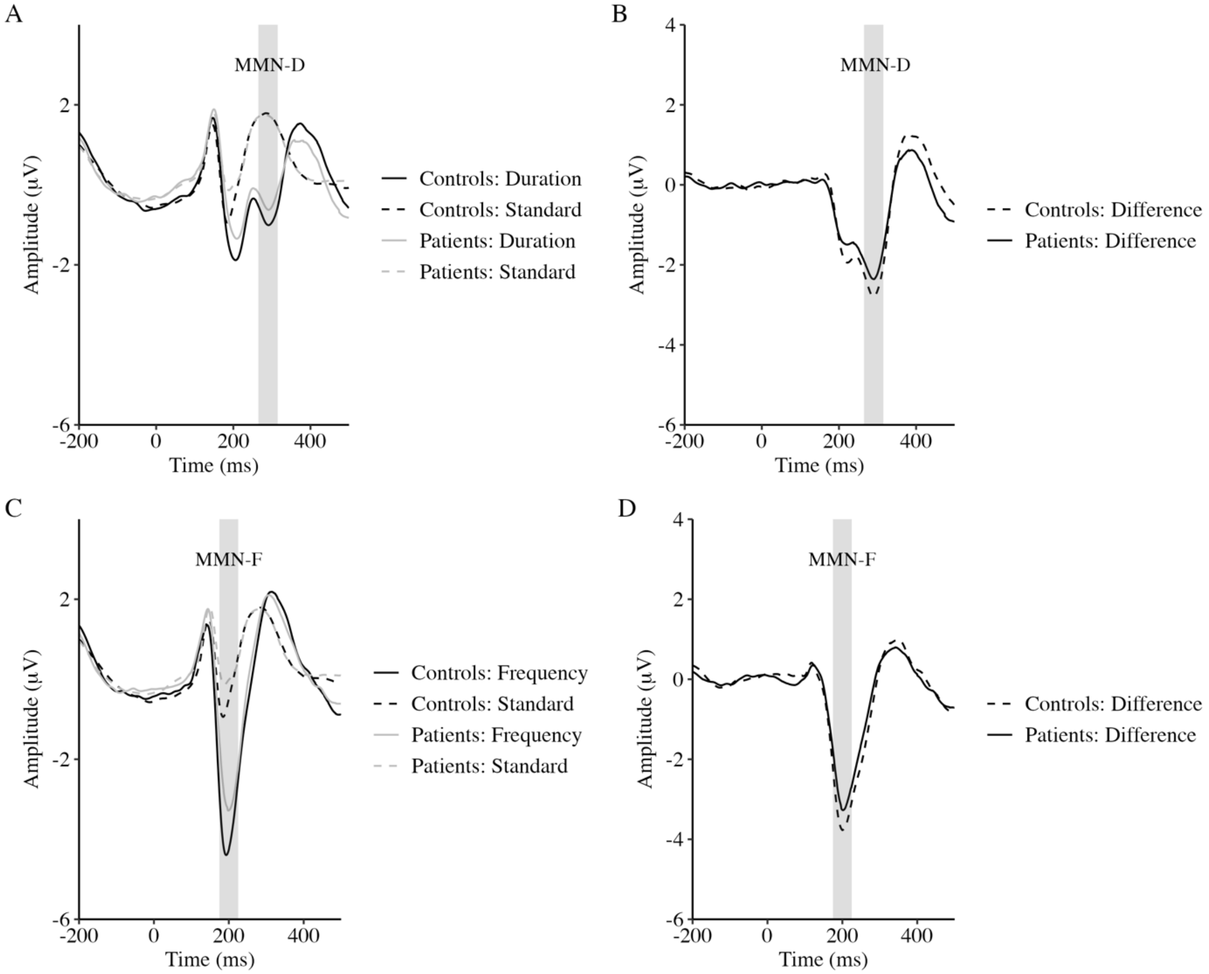

Supplementary Figure 3

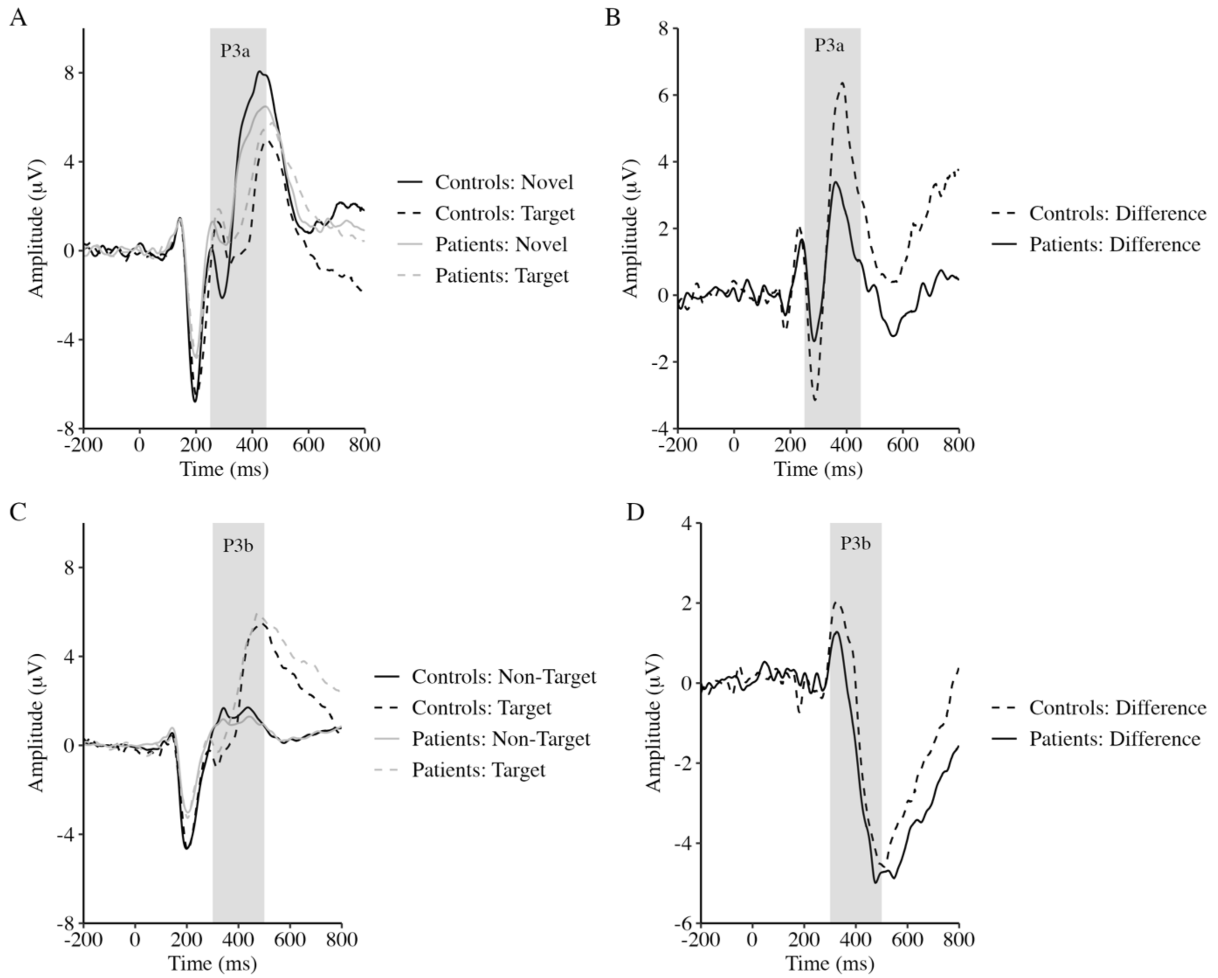

Supplementary Figure 4

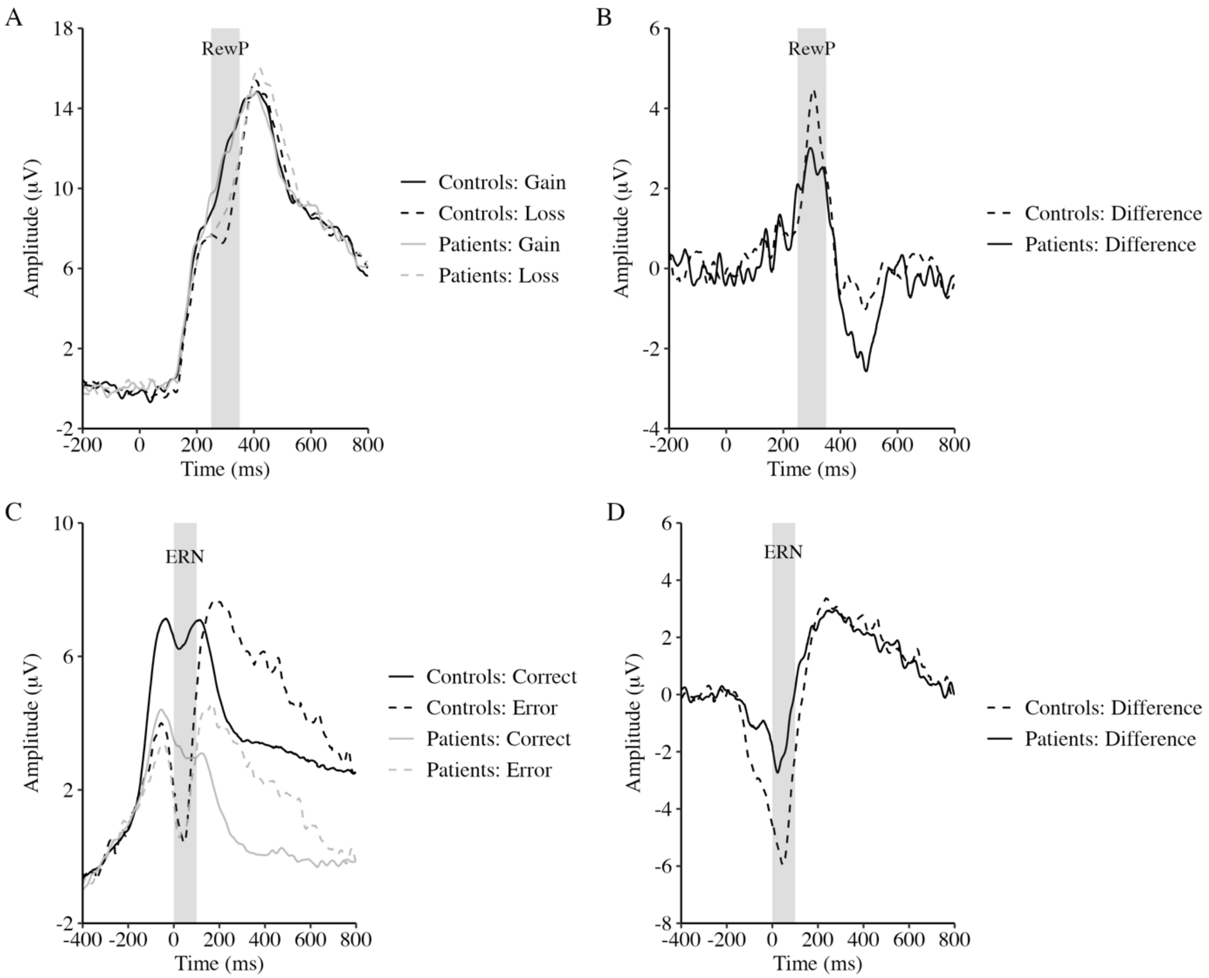

Supplementary Table 1

Summary Information for Event-Related Potential (μV) Data

	Patients		Controls	
	M	*SD*	*M*	*SD*
MMN-D				
Standard	1.62	1.2	1.71	2.03
Deviant	-0.47	2.08	-0.83	2.98
Deviant-Standard	-2.09	1.69	-2.54	1.86
MMN-F				
Standard	-0.01	1.34	-0.4	1.98
Deviant	-2.81	2.06	-3.73	2.93
Deviant-Standard	-2.8	1.63	-3.33	1.72
P3a				
Target	2.04	4.08	1.31	3.74
Novel	3.51	4.27	3.54	4.27
Novel-Target	1.47	4	2.23	3.41
P3b				
Target	2.66	4.22	2.31	3.63
Nontarget	1.14	2.04	1.38	2.03
Target-Nontarget	-1.52	4.25	-0.93	3.35
ERN				
Correct	2.77	5.56	6.51	7.5
Error	1.05	4.99	2.2	6.93
Error-Correct	-1.72	4.85	-4.31	7.53
RewP				
Loss	9.11	7	8.21	5.9
Gain	11.35	7.8	11.34	7.21
Gain-Loss	2.24	4.05	3.13	5.26
P3a collapsed localizer				
Target	4.73	5.23	3.91	4.91
Novel	6.15	4.73	7.64	5.08
Novel-Target	1.42	4.66	3.73	4.62
P3b collapsed localizer				
Target	5.3	5.29	5.25	4.59
Nontarget	1.15	2.31	1.25	2.23
Target-Nontarget	-4.15	5.32	-4	4.4
Post-error Slowing				
Congruent	632.51	164.07	547.39	114.2
Incongruent	616.61	155.86	550.22	129.52
Incongruent-Congruent	-15.24	93.9	2.82	82.98

Note: MMN-D = mismatch negativity duration deviant; MMN-F = mismatch negativity frequency deviant; ERN = error-related negativity; RewP= reward positivity.

Supplementary Table 2

Demographic and Diagnostic Summary for Participants included in Mismatch Negativity (MMN) Analyses

	Patients (n=145)		Controls (n=169)	
	M	*SD*	*M*	*SD*
Age	52.6	8.53	56.7	8.97
Sex	n	%	n	%
Female	62	42.80%	78	46.20%
Male	83	57.20%	91	53.80%
Race				
White	121	83.40%	154	91.10%
Black/African American	19	13.10%	10	5.62%
Asian	4	2.76%	0	0.00%
More than one race	0	0.00%	2	1.18%
Unknown	1	0.69%	3	1.78%
Diagnosis	Lifetime	Past-month	Lifetime	Past-month
Schizophrenia	52 (35.86%)	51 (35.17%)	0 (0%)	0 (0%)
Schizoaffective Disorder	20 (13.79%)	19 (13.1%)	0 (0%)	0 (0%)
Bipolar I	48 (33.1%)	7 (4.83%)	0 (0%)	0 (0%)
Bipolar II	1 (0.69%)	0 (0%)	1 (0.59%)	1 (0.59%)
Other Bipolar[a]	4 (2.76%)	1 (0.69%)	0 (0%)	0 (0%)
Delusional Disorder	1 (0.69%)	1 (0.69%)	0 (0%)	0 (0%)
Substance Induced Psychotic Disorder	6 (4.14%)	0 (0%)	0 (0%)	0 (0%)
Psychotic Disorder NOS	6 (4.14%)	0 (0%)	0 (0%)	0 (0%)
Major Depressive Disorder[b]	13 (8.97%)	4 (2.76%)	34 (20.12%)	6 (3.55%)
Depressive Disorder NOS[c]	6 (4.14%)	1 (0.69%)	6 (3.55%)	1 (0.59%)
Substance Induced Mood Disorder	0 (0.00%)	0 (0%)	0 (0%)	0 (0%)
Dysthymic Disorder	1 (0.69%)	1 (0.69%)	5 (2.96%)	5 (2.96%)
BPRS	*M*	*SD*	*M*	*SD*
Affect	8.00	4.05	6.65	3.08
Positive Symptoms	7.26	4.44	4.29	0.82
Negative Symptoms	6.52	3.16	4.59	1.28
Resistance	5.54	3.16	3.70	1.41
Activation	4.19	1.74	3.40	0.94
SAPS/SANS	*M*	*SD*	*M*	*SD*
Thought Disorder	5.42	5.67	1.7	2.73
Reality Distortion	3.33	5.4	0.06	0.32
Apathy/Asociality	12.9	7.67	4.84	5.96
Inexpressivity	7.79	8.91	1.82	3.44

Note: Demographic and diagnostic summary statistics are reported for 314 participants included in group-level analyses. BPRS and SAPS/SANS statistics are reported for 295 participants included in symptom-level analyses. NOS = Not other specified. BPRS =Brief Psychiatric Rating Scale; SAPS/SANS =Scale for the Assessment of Positive and Negative Symptoms.

Supplementary Table 3

Demographic and Diagnostic Summary for Participants included in P3a and P3b Analyses

	Patients (n=149)		Controls (n=166)	
	M	*SD*	*M*	*SD*
Age	52.60	8.50	57.00	8.94
Sex	n	%	n	%
Female	68	45.60%	77	46.40%
Male	81	54.40%	89	53.60%
Race				
White	125	83.90%	153	92.20%
Black/African American	20	13.40%	9	5.42%
Asian	3	2.01%	0	0.00%
More than one race	0	0.00%	1	0.60%
Unknown	1	0.67%	3	1.81%
Diagnosis	Lifetime	Past-month	Lifetime	Past-month
Schizophrenia	53 (35.57%)	52 (34.9%)	0 (0%)	0 (0%)
Schizoaffective Disorder	20 (13.42%)	20 (13.42%)	0 (0%)	0 (0%)
Bipolar I	46 (30.87%)	7 (4.7%)	0 (0%)	0 (0%)
Bipolar II	1 (0.67%)	0 (0%)	1 (0.67%)	1 (0.67%)
Other Bipolar[a]	5 (3.36%)	1 (0.67%)	0 (0%)	0 (0%)
Delusional Disorder	1 (0.67%)	1 (0.67%)	0 (0%)	0 (0%)
Substance Induced Psychotic Disorder	7 (4.7%)	0 (0%)	0 (0%)	0 (0%)
Psychotic Disorder NOS	7 (4.7%)	0 (0%)	0 (0%)	0 (0%)
Major Depressive Disorder[b]	14 (9.4%)	5 (3.36%)	35 (23.49%)	6 (4.03%)
Depressive Disorder NOS[c]	7 (4.7%)	1 (0.67%)	5 (3.36%)	1 (0.67%)
Substance Induced Mood Disorder	1 (0.67%)	0 (0%)	0 (0%)	0 (0%)
Dysthymic Disorder	2 (1.34%)	2 (1.34%)	5 (3.36%)	5 (3.36%)
BPRS	*M*	*SD*	*M*	*SD*
Affect	8.18	4.15	6.58	3.04
Positive Symptoms	6.97	4.14	4.27	0.80
Negative Symptoms	6.46	3.07	4.58	1.28
Resistance	5.37	2.97	3.66	1.38
Activation	4.20	1.80	3.42	0.95
SAPS/SANS	*M*	*SD*	*M*	*SD*
Thought Disorder	4.93	5.63	1.69	2.70
Reality Distortion	3.21	5.24	0.06	0.33
Apathy/Asociality	13.35	7.73	4.71	5.87
Inexpressivity	7.53	8.73	1.66	3.12

Note: Demographic and diagnostic summary statistics are reported for 315 participants included in group-level analyses. BPRS and SAPS/SANS statistics are reported for 295 participants included in symptom-level analyses. NOS = Not other specified. BPRS =Brief Psychiatric Rating Scale; SAPS/SANS =Scale for the Assessment of Positive and Negative Symptoms.

Supplementary Table 4

Demographic and Diagnostic Summary for Participants included in Error-Related Negativity (ERN) Analyses

	Patients (n=135)		Controls (n=150)	
	M	*SD*	*M*	*SD*
Age	52.3	8.49	57.3	8.88
Sex	n	%	n	%
Female	58	43.00%	67	44.70%
Male	77	57.00%	83	55.30%
Race				
White	112	83.00%	137	91.30%
Black/African American	18	13.30%	9	6.00%
Asian	4	2.96%	0	0.00%
More than one race	0	0.00%	1	0.67%
Unknown	1	0.74%	3	2.00%
Diagnosis	Lifetime	Past-month	Lifetime	Past-month
Schizophrenia	45 (33.33%)	44 (32.59%)	0 (0%)	0 (0%)
Schizoaffective Disorder	19 (14.07%)	19 (14.07%)	0 (0%)	0 (0%)
Bipolar I	47 (34.81%)	6 (4.44%)	0 (0%)	0 (0%)
Bipolar II	1 (0.74%)	0 (0%)	1 (0.67%)	1 (0.67%)
Other Bipolar[a]	4 (2.96%)	1 (0.74%)	0 (0%)	0 (0%)
Delusional Disorder	0 (0%)	0 (0%)	0 (0%)	0 (0%)
Substance Induced Psychotic Disorder	6 (4.44%)	0 (0%)	0 (0%)	0 (0%)
Psychotic Disorder NOS	6 (4.44%)	0 (0%)	0 (0%)	0 (0%)
Major Depressive Disorder[b]	12 (8.89%)	4 (2.96%)	32 (21.33%)	6 (4%)
Depressive Disorder NOS[c]	3 (2.22%)	0 (0%)	2 (1.33%)	1 (0.67%)
Substance Induced Mood Disorder	1 (0.74%)	0 (0%)	0 (0%)	0 (0%)
Dysthymic Disorder	1 (0.74%)	1 (0.74%)	4 (2.67%)	4 (2.67%)
BPRS	*M*	*SD*	*M*	*SD*
Affect	8.04	4.00	6.63	3.10
Positive Symptoms	7.27	4.45	4.25	0.80
Negative Symptoms	6.51	3.17	4.65	1.35
Resistance	5.56	3.11	3.61	1.15
Activation	4.24	1.76	3.43	0.97
SAPS/SANS	*M*	*SD*	*M*	*SD*
Thought Disorder	5.35	5.57	1.74	2.68
Reality Distortion	3.28	5.30	0.06	0.34
Apathy/Asociality	13.05	7.82	5.01	6.03
Inexpressivity	7.73	9.03	1.92	3.55

Note: Demographic and diagnostic summary statistics are reported for 285 participants included in group-level analyses. BPRS and SAPS/SANS statistics are reported for 269 participants included in symptom-level analyses. NOS = Not other specified. BPRS =Brief Psychiatric Rating Scale; SAPS/SANS =Scale for the Assessment of Positive and Negative Symptoms.

Supplementary Table 5

Demographic and Diagnostic Summary for Participants included in Reward Positivity (RewP) Analyses

	Patients (n=124)		Controls (n=151)	
	M	*SD*	*M*	*SD*
Age	52.8	8.68	56.9	8.94
Sex	n	%	n	%
Female	55	44.40%	67	44.40%
Male	69	55.60%	84	55.60%
Race				
White	107	86.30%	139	92.00%
Black/African American	14	11.30%	8	5.30%
Asian	3	2.42%	0	0.00%
More than one race	0	0.00%	1	0.66%
Unknown	0	0.00%	3	1.99%
Diagnosis	Lifetime	Past-month	Lifetime	Past-month
Schizophrenia	43 (34.68%)	42 (33.87%)	0 (0%)	0 (0%)
Schizoaffective Disorder	13 (10.48%)	13 (10.48%)	0 (0%)	0 (0%)
Bipolar I	44 (35.48%)	6 (4.84%)	0 (0%)	0 (0%)
Bipolar II	1 (0.81%)	0 (0%)	1 (0.66%)	1 (0.66%)
Other Bipolar[a]	3 (2.42%)	0 (0%)	0 (0%)	0 (0%)
Delusional Disorder	1 (0.81%)	1 (0.81%)	0 (0%)	0 (0%)
Substance Induced Psychotic Disorder	6 (4.84%)	0 (0%)	0 (0%)	0 (0%)
Psychotic Disorder NOS	6 (4.84%)	0 (0%)	0 (0%)	0 (0%)
Major Depressive Disorder[b]	9 (7.26%)	3 (2.42%)	33 (21.85%)	6 (3.97%)
Depressive Disorder NOS[c]	6 (4.84%)	1 (0.81%)	3 (1.99%)	1 (0.66%)
Substance Induced Mood Disorder	0 (0%)	0 (0%)	0 (0%)	0 (0%)
Dysthymic Disorder	1 (0.81%)	1 (0.81%)	5 (3.31%)	5 (3.31%)
BPRS	*M*	*SD*	*M*	*SD*
Affect	7.98	4.05	6.51	3.06
Positive Symptoms	6.94	4.15	4.25	0.80
Negative Symptoms	6.43	2.90	4.61	1.34
Resistance	5.46	3.11	3.63	1.27
Activation	4.03	1.52	3.43	0.96
SAPS/SANS	*M*	*SD*	*M*	*SD*
Thought Disorder	4.91	5.36	1.70	2.66
Reality Distortion	3.11	4.87	0.04	0.29
Apathy/Asociality	12.74	7.92	5.00	6.05
Inexpressivity	7.99	9.15	1.79	3.29

Note: Demographic and diagnostic summary statistics are reported for 275 participants included in group-level analyses. BPRS and SAPS/SANS statistics are reported for 259 participants included in symptom-level analyses. NOS = Not other specified. BPRS =Brief Psychiatric Rating Scale; SAPS/SANS =Scale for the Assessment of Positive and Negative Symptoms.

Supplementary Table 6

Reliability and Data Quality Summary Information for Controls

ERP	Event	Group-Level Reliability	Subject-Level Reliability	Between-Person σ	Within-Person σ	ICC	SME
MMN-D	Standard	0.97 (0.97, 0.98)	0.96 (0.07)	2.02 (1.81, 2.26)	15.08 (15.05, 15.12)	0.02 (0.01, 0.02)	0.36
	Deviant	0.89 (0.86, 0.91)	0.82 (0.10)	2.82 (2.49, 3.18)	15.48 (15.38, 15.59)	0.03 (0.03, 0.04)	1.07
	Difference	0.68 (0.61, 0.75)				0.01 (0.004, 0.01)	
MMN-F	Standard	0.98 (0.97, 0.98)	0.97 (0.05)	1.97 (1.77, 2.20)	13.42 (13.39, 13.46)	0.02 (0.02, 0.03)	0.33
	Deviant	0.89 (0.86, 0.91)	0.89 (0.10)	2.78 (2.46, 3.14)	14.76 (14.65, 14.86)	0.03 (0.03, 0.04)	1.01
	Difference	0.65 (0.57, 0.72)				0.01 (0.004, 0.01)	
P3a	Target	0.59 (0.49, 0.67)	0.64 (0.09)	2.89 (2.37, 3.45)	11.66 (11.40, 11.93)	0.06 (0.04, 0.08)	2.42
	Novel	0.68 (0.60, 0.75)	0.69 (0.10)	3.55 (3.01, 4.16)	11.75 (11.49, 12.03)	0.08 (0.06, 0.11)	2.47
	Difference	0.34 (0.20, 0.48)				0.02 (0.01,	
P3b	Target	0.61 (0.53, 0.70)	0.63 (0.09)	2.86 (2.37, 3.38)	10.85 (10.60, 11.10)	0.07 (0.05, 0.09)	2.32
	Nontarget	0.83 (0.80, 0.87)	0.83 (0.09)	1.85 (1.62, 2.11)	11.22 (11.13, 11.30)	0.03 (0.02, 0.03)	0.85
	Difference	0.64 (0.55, 0.71)				0.04 (0.03, 0.06)	
ERN	Correct	0.79 (0.72, 0.85)	0.98 (0.08)	9.27 (7.68, 11.12)	21.01 (20.32, 21.72)	0.16 (0.12, 0.22)	1.41
	Error	0.83 (0.78, 0.87)	0.83 (0.10)	6.15 (5.31, 7.11)	15.80 (15.48, 16.13)	0.13 (0.10, 0.17)	2.89
	Difference	0.81 (0.76, 0.85)				0.05 (0.04, 0.06)	
RewP	Gain	0.86 (0.83, 0.90)	0.86 (0.07)	6.71 (5.87, 7.68)	11.22 (10.92, 11.53)	0.26 (0.21, 0.32)	2.75
	Loss	0.81 (0.77, 0.85)	0.82 (0.07)	5.37 (4.66, 6.16)	10.87 (10.58, 11.17)	0.20 (0.15, 0.24)	2.56
	Difference	0.50 (0.38, 0.61)				0.05 (0.03, 0.08)	
P3a cl	Target	0.67 (0.60, 0.74)	0.72 (0.09)	4.09 (3.46, 4.78)	13.64 (13.34, 13.96)	0.08 (0.06, 0.11)	2.82
	Novel	0.70 (0.63, 0.76)	0.69 (0.09)	4.29 (3.65, 5.00)	13.54 (13.23, 13.85)	0.09 (0.07, 0.12)	2.85
	Difference	0.34 (0.20, 0.48)				0.02 (0.01, 0.04)	
P3b cl	Target	0.70 (0.63, 0.76)	0.71 (0.08)	3.88 (3.30, 4.52)	12.23 (11.96, 12.51)	0.09 (0.07, 0.12)	2.6
	Nontarget	0.84 (0.80, 0.87)	0.81 (0.09)	2.04 (1.79, 2.32)	12.27 (12.17, 12.37)	0.03 (0.02, 0.03)	0.93
	Difference	0.64 (0.55, 0.71)				0.04 (0.03, 0.06)	
PES	Correct	0.93 (0.91, 0.95)	0.90 (0.08)	108.76 (92.90, 127.42)	131.10 (126.87, 135.53)	0.41 (0.33, 0.49)	

Note: Group- and subject-level reliability is presented as dependability (f). ICC = intraclass correlation coefficient; SME = Standard measurement error. MMN-D = Mismatch Negativity Duration; MMN-F: Mismatch Negativity Frequency; ERN = Error-related Negativity; RewP = Reward Positivity; P3a cl = P3a collapsed localizer; P3b = P3b collapsed localizer; PES = post-error slowing.

Supplementary Table 7

Reliability and Data Quality Summary Information for Patients

ERP	Event	Group-Level Reliability	Subject-Level Reliability	Between-Person σ	Within-Person σ	ICC	SME
MMN-D	Standard	0.93 (0.91, 0.94)	0.93 (0.08)	1.16 (1.02, 1.31)	13.94 (13.91, 13.98)	0.01 (0.01, 0.01)	0.39
	Deviant	0.78 (0.73, 0.83)	0.81 (0.10)	1.79 (1.53, 2.08)	14.23 (14.13, 14.34)	0.02 (0.01, 0.02)	1.22
	Difference	0.64 (0.55, 0.72)				0.004 (0.003, 0.01)	
MMN-F	Standard	0.95 (0.94, 0.96)	0.95 (0.07)	1.32 (1.17, 1.50)	12.80 (12.76, 12.83)	0.01 (0.01, 0.01)	0.34
	Deviant	0.84 (0.80, 0.87)	0.84 (0.09)	1.90 (1.65, 2.18)	12.47 (12.37, 12.56)	0.02 (0.02, 0.03)	0.99
	Difference	0.71 (0.64, 0.77)				0.01 (0.004, 0.01)	
P3a	Target	0.67 (0.58, 0.74)	0.68 (0.09)	3.37 (2.81, 3.97)	11.52 (11.25, 11.80)	0.08 (0.06, 0.11)	2.37
	Novel	0.76 (0.70, 0.81)	0.74 (0.08)	3.70 (3.15, 4.30)	10.63 (10.39, 10.88)	0.11 (0.08, 0.14)	2.2
	Difference	0.50 (0.38, 0.60)				0.04 (0.02, 0.06)	
P3b	Target	0.66 (0.58, 0.73)	0.65 (0.10)	3.50 (2.92, 4.12)	12.06 (11.77, 12.36)	0.08 (0.06, 0.11)	2.45
	Nontarget	0.84 (0.80, 0.87)	0.80 (0.10)	1.86 (1.62, 2.14)	11.98 (11.88, 12.07)	0.02 (0.02, 0.03)	0.89
	Difference	0.70 (0.62, 0.76)				0.05 (0.04, 0.07)	
ERN	Correct	0.97 (0.97, 0.98)	0.97 (0.09)	5.52 (4.87, 6.26)	14.16 (14.06, 14.27)	0.13 (0.11, 0.16)	1.73
	Error	0.51 (0.40, 0.62)	0.78 (0.20)	3.60 (2.87, 4.42)	27.49 (27.08, 27.91)	0.02 (0.01, 0.03)	2.42
	Difference	0.34 (0.18, 0.50)				0.01 (0.003, 0.01)	
RewP	Gain	0.89 (0.86, 0.92)	0.88 (0.04)	7.47 (6.49, 8.61)	10.98 (10.65, 11.32)	0.32 (0.26, 0.38)	2.63
	Loss	0.87 (0.83, 0.90)	0.86 (0.06)	6.62 (5.70, 7.65)	10.89 (10.56, 11.23)	0.27 (0.21, 0.33)	2.68
	Difference	0.26 (0.11, 0.43)				0.02 (0.01, 0.04)	
P3a cl	Target	0.73 (0.67, 0.79)	0.75 (0.08)	4.54 (3.86, 5.28)	13.23 (12.91, 13.55)	0.11 (0.08, 0.14)	2.72
	Novel	0.75 (0.68, 0.80)	0.73 (0.08)	4.09 (3.47, 4.76)	12.12 (11.86, 12.40)	0.10 (0.08, 0.13)	2.5
	Difference	0.50 (0.38, 0.60)				0.04 (0.02, 0.06)	
P3b cl	Target	0.72 (0.65, 0.78)	0.72 (0.1)	4.58 (3.89, 5.35)	13.73 (13.40, 14.05)	0.10 (0.07, 0.13)	2.8
	Nontarget	0.84 (0.81, 0.88)	0.78 (0.1)	2.12 (1.84, 2.44)	13.21 (13.11, 13.31)	0.03 (0.02, 0.03)	0.97
	Difference	0.70 (0.62, 0.76)				0.05 (0.04, 0.07)	
PES	Correct	0.93 (0.91, 0.95)	0.93 (0.04)	150.63 (129.08, 176.38)	197.27 (191.62, 203.08)	0.37 (0.30, 0.45)	

Note: Group- and subject-level reliability is presented as dependability (f). ICC = intraclass correlation coefficient; SME = Standard measurement error. MMN-D = Mismatch Negativity Duration; MMN-F: Mismatch Negativity Frequency; ERN = Error-related Negativity; RewP = Reward Positivity; P3a cl = P3a collapsed localizer; P3b cl = P3b collapsed localizer; PES = post-error slowing.

Supplementary Table 8

Summary Information for Location-Only and Location-Scale Model Fits

	MMN-D			MMN-F	
Model	$\widehat{\text{elpd}}_{\text{loo}}$	$\text{SE}(\widehat{\text{elpd}}_{\text{loo}})$	Model	$\widehat{\text{elpd}}_{\text{loo}}$	$\text{SE}(\widehat{\text{elpd}}_{\text{loo}})$
Location-Only	-2,760,619	5,404.36	Location-Only	-2,677,968	4,483.34
Location-Scale	-2,639,254	733.43	Location-Scale	-2,582,003	721.88
	P3a			P3b	
Model	$\widehat{\text{elpd}}_{\text{loo}}$	$\text{SE}(\widehat{\text{elpd}}_{\text{loo}})$	Model	$\widehat{\text{elpd}}_{\text{loo}}$	$\text{SE}(\widehat{\text{elpd}}_{\text{loo}})$
Location-Only	-58,722.82	381.80	Location-Only	-271,587.1	1,150.52
Location-Scale	-57,551.60	114.52	Location-Scale	-261,903.7	253.34
	ERN			RewP	
Model	$\widehat{\text{elpd}}_{\text{loo}}$	$\text{SE}(\widehat{\text{elpd}}_{\text{loo}})$	Model	$\widehat{\text{elpd}}_{\text{loo}}$	$\text{SE}(\widehat{\text{elpd}}_{\text{loo}})$
Location-Only	-409,170.0	4,071.98	Location-Only	-37,764.30	99.87
Location-Scale	-352,649.6	302.74	Location-Scale	-37,321.74	88.01
	P3a collapsed localizer			P3b collapsed localizer	
Model	$\widehat{\text{elpd}}_{\text{loo}}$	$\text{SE}(\widehat{\text{elpd}}_{\text{loo}})$	Model	$\widehat{\text{elpd}}_{\text{loo}}$	$\text{SE}(\widehat{\text{elpd}}_{\text{loo}})$
Location-Only	-60,913.67	388.46	Location-Only	-278,442.0	1,241.70
Location-Scale	-59,749.00	114.58	Location-Scale	-268,199.8	253.56

Note: Log predictive density = ($\widehat{elpd}_{loo}$); Standard error of log predictive density = $\text{SE}(\widehat{\text{elpd}}_{\text{loo}})$. RewP = Reward Positivity; ERN = Error-related Negativity; MMN-D = Mismatch Negativity Duration; MMN-F: Mismatch Negativity Frequency

Supplementary Table 9

Estimates from Location-Scale Multilevel Model Predicting P3a Collapsed Localizer Amplitude

Predictor	Estimate	*SE*	95% CrI
Location Portion			
Target: Controls (Intercept)	3.94	0.37	3.21, 4.67
Target: Patients	0.80	0.53	-0.24, 1.84
Novel: Controls	3.54	0.33	2.88, 4.19
Novel: Patients	-2.14	0.46	-3.05, -1.24
Scale Portion (*SD*)			
Target: Controls (Intercept)	2.50	0.02	2.45, 2.54
Target: Patients	-0.03	0.03	-0.09, 0.03
Novel: Controls	0.01	0.02	-0.03, 0.05
Novel: Patients	-0.02	0.03	-0.08, 0.04
Random Effects (*SD*)			
Mean Structure			
Target (Intercept)	4.10	0.23	3.66, 4.58
Novel	2.36	0.32	1.73, 2.96
Variance Structure			
Target (Intercept)	0.25	0.01	0.22, 0.28
Novel	0.16	0.02	0.13, 0.20

Note: Estimates of parameters represent the median, and parameters in standard deviations (*SD*) units are shown on a log scale. *SE* = standard error; 95% CrI = 95% credible interval

Supplementary Table 10

Pairwise Contrasts for Group-Related Differences

Location Portion of Models					
P3a collapsed localizer			P3b collapsed localizer		
Parameter	Median	95% CrI	Parameter	Median	95% CrI
Target	0.80	(-0.24, 1.84)	Target	0.19	(-0.78, 1.15)
Novel	**-1.34**	**(-2.39, -0.30)**	Nontarget	-0.18	(-0.60, 0.23)
Novel-Target	**-2.13**	**(-3.05, -1.24)**	Target-Nontarget	-0.38	(-1.27, 0.53)
**Scale Portion of Models**					
P3a collapsed localizer			P3b collapsed localizer		
Parameter	Median	95% CrI	Parameter	Median	95% CrI
Target	-0.03	(-0.09, 0.03)	Target	-0.01	(-0.08, 0.06)
Novel	-0.05	(-0.11, 0.01)	Nontarget	0.002	(-0.06, 0.06)
Novel-Target	-0.02	(-0.08, 0.04)	Target-Nontarget	0.01	(-0.03, 0.06)

Note: All estimates are for the patient minus control contrasts. If the 95% CrI of the contrast excludes zero, this is interpreted as evidence of a difference. In such instances, the contrast is shown in bold font.

Supplementary Table 11

Estimates from Location-Scale Multilevel Model Predicting P3b Collapsed Localizer Amplitude

Predictor	Estimate	*SE*	95% CrI
Location Portion			
Target: Controls (Intercept)	5.20	0.34	4.45, 5.88
Target: Patients	0.19	0.49	-0.78, 1.15
Nontarget: Controls	-3.97	0.32	-4.60, -3.35
Nontarget: Patients	-0.37	0.46	-1.27, 0.53
Scale Portion (*SD*)			
Target: Controls (Intercept)	2.46	0.02	2.41, 2.51
Target: Patients	-0.01	0.03	-0.08, 0.06
Nontarget: Controls	-0.07	0.02	-0.10, -0.03
Nontarget: Patients	0.01	003	-0.03, 0.06
Random Effects (*SD*)			
Mean Structure			
Target (Intercept)	3.75	0.21	3.35, 4.18
Nontarget	3.31	0.21	2.91, 3.74
Variance Structure			
Target (Intercept)	0.27	0.01	0.24, 0.30
Nontarget	0.16	0.01	0.13, 0.18

Note: Estimates of parameters represent the median, and parameters in standard deviations (*SD*) units are shown on a log scale. *SE* = standard error; 95% CrI = 95% credible interval

Supplementary Table 12

Parameter Estimates and Simple Slopes for MMN Duration-Symptom Models

Location Portion						
Subscale	Intercept	Event	Symptom	Event x Symptom	Standard Slope	Deviant Slope
BPRS						
Activation	1.58 (1.14, 2.04)	**-2.63 (-3.17, -2.07)**	0.02 (-0.10, 0.13)	0.05 (-0.08, 0.19)	0.02 (-0.10, 0.13)	0.07 (-0.10, 0.24)
Affect	1.99 (1.63, 2.37)	**-2.32 (-2.76, -1.88)**	**-0.05 (-0.09, -0.004)**	-0.01 (-0.07, 0.04)	**-0.05 (-0.09, -0.004)**	-0.06 (-0.13, 0.002)
Neg. Symptoms	1.42 (1.04, 1.82)	**-2.63 (-3.09, -2.16)**	0.04 (-0.03, 0.10)	0.04 (-0.04, 0.12)	0.04 (-0.03, 0.10)	0.08 (-0.02, 0.18)
Pos. Symptoms	1.64 (1.33, 1.96)	**-2.73 (-3.11, -2.36)**	0.001 (-0.05, 0.05)	0.05 (-0.002, 0.11)	0.001 (-0.05, 0.05)	0.06 (-0.02, 0.13)
Resistance	1.63 (1.31, 1.96)	**-2.43 (-2.83, -2.03)**	0.002 (-0.06, 0.06)	0.001 (-0.08, 0.08)	0.002 (-0.06, 0.06)	0 (-0.09, 0.10)
SAPS/SANS						
Inexpressivity	1.59 (1.40, 1.79)	**-2.56 (-2.79, -2.33)**	0.01 (-0.01, 0.03)	**0.03 (0.002, 0.06)**	0.01 (-0.01, 0.03)	**0.04 (0.01, 0.08)**
Reality Distortion	1.63 (1.46, 1.81)	**-2.47 (-2.67, -2.26)**	0.004 (-0.04, 0.04)	0.03 (-0.02, 0.08)	0.004 (-0.04, 0.04)	0.03 (-0.03, 0.09)
Apathy/Asociality	1.66 (1.42, 1.89)	**-2.84 (-3.11, -2.56)**	-0.002 (-0.02, 0.02)	**0.05 (0.03, 0.07)**	-0.002 (-0.02, 0.02)	**0.05 (0.02, 0.08)**
Thought Disorder	1.62 (1.43, 1.82)	**-2.58 (-2.81, -2.35)**	0.01 (-0.03, 0.04)	**0.04 (0.004, 0.09)**	0.01 (-0.03, 0.04)	**0.05 (0.002, 0.10)**
Scale Portion						
Subscale	Intercept	Event	Symptom	Event x Symptom	Standard Slope	Deviant Slope
BPRS						
Activation	2.42 (2.32, 2.50)	**0.02 (0.01, 0.04)**	**0.02 (0.002, 0.05)**	**-0.01 (-0.01, -0.001)**	**0.02 (0.002, 0.05)**	0.02 (-0.003, 0.04)
Affect	2.53 (2.46, 2.60)	0.01 (-0.003, 0.03)	-0.003 (-0.01, 0.01)	-0.001 (-0.002, 0.001)	-0.003 (-0.01, 0.01)	-0.004 (-0.01, 0.01)
Neg. Symptoms	2.47 (2.39, 2.54)	0.01 (-0.01, 0.02)	0.01 (-0.01, 0.02)	-0.00001 (-0.003, 0.003)	0.01 (-0.01, 0.02)	0.01 (-0.01, 0.02)
Pos. Symptoms	2.47 (2.41, 2.54)	**0.01 (0.001, 0.03)**	0.01 (-0.003, 0.02)	-0.001 (-0.003, 0.001)	0.01 (-0.003, 0.02)	0.005 (-0.01, 0.01)
Resistance	2.49 (2.42, 2.55)	0.01 (-0.003, 0.02)	0.004 (-0.01, 0.02)	-0.001 (-0.004, 0.001)	0.004 (-0.01, 0.02)	0.003 (-0.01, 0.02)
SAPS/SANS						
Inexpressivity	2.49 (2.45, 2.53)	0.01 (-0.001, 0.01)	0.004 (-0.0003, 0.01)	-0.0002 (-0.001, 0.001)	0.004 (-0.0003, 0.01)	0.004 (-0.001, 0.01)
Reality Distortion	2.5 (2.47, 2.54)	0.01 (-0.00001, 0.01)	0.004 (-0.004, 0.01)	-0.0009 (-0.002, 0.001)	0.004 (-0.004, 0.01)	0.003 (-0.01, 0.01)
Apathy/Asociality	2.49 (2.44, 2.53)	**0.01 (0.001, 0.02)**	0.002 (-0.002, 0.01)	-0.0006 (-0.001, 0.0002)	0.002 (-0.002, 0.01)	0.002 (-0.002, 0.01)
Thought Disorder	2.49 (2.45, 2.53)	**0.01 (0.004, 0.02)**	0.01 (-0.00002, 0.01)	**-0.002 (-0.003, -0.001)**	0.01 (-0.00002, 0.01)	0.005 (-0.002, 0.01)

Note: Instances where the 95% CrI does not contain zero are bolded for ease of interpretation.

Supplementary Table 13

Parameter Estimates and Simple Slopes for MMN Frequency-Symptom Models

Location Portion						
Subscale	Intercept	Event	Symptom	Event x Symptom	Standard Slope	Deviant Slope
BPRS						
Activation	-0.57 (-1.07, -0.04)	**-3.69 (-4.24, -3.14)**	0.08 (-0.05, 0.21)	**0.14 (0.01, 0.28)**	0.08 (-0.05, 0.21)	**0.23 (0.02, 0.42)**
Affect	-0.01 (-0.40, 0.40)	**-3.2 (-3.64, -2.77)**	-0.03 (-0.09, 0.02)	0.01 (-0.05, 0.06)	-0.03 (-0.09, 0.02)	-0.03 (-0.10, 0.05)
Neg. Symptoms	-0.77 (-1.21, -0.33)	**-3.19 (-3.66, -2.73)**	**0.09 (0.02, 0.17)**	0.01 (-0.07, 0.09)	**0.09 (0.02, 0.17)**	0.1 (-0.01, 0.22)
Pos. Symptoms	-0.35 (-0.71, 0.003)	**-3.38 (-3.75, -3.01)**	0.02 (-0.04, 0.07)	0.04 (-0.02, 0.10)	0.02 (-0.04, 0.07)	0.06 (-0.02, 0.14)
Resistance	-0.25 (-0.63, 0.12)	**-3.26 (-3.64, -2.86)**	0.0002 (-0.07, 0.07)	0.02 (-0.05, 0.10)	0 (-0.07, 0.07)	0.02 (-0.09, 0.13)
SAPS/SANS						
Inexpressivity	-0.44 (-0.65, -0.23)	**-3.2 (-3.42, -2.97)**	**0.04 (0.02, 0.07)**	0.01 (-0.02, 0.04)	**0.04 (0.02, 0.07)**	**0.05 (0.01, 0.09)**
Reality Distortion	-0.26 (-0.46, -0.07)	**-3.17 (-3.38, -2.96)**	0.01 (-0.04, 0.06)	0.01 (-0.04, 0.06)	0.01 (-0.04, 0.06)	0.02 (-0.05, 0.09)
Apathy/Asociality	-0.52 (-0.78, -0.24)	**-3.46 (-3.74, -3.19)**	**0.03 (0.01, 0.05)**	**0.04 (0.01, 0.06)**	**0.03 (0.01, 0.05)**	**0.07 (0.03, 0.10)**
Thought Disorder	-0.36 (-0.59, -0.12)	**-3.3 (-3.54, -3.07)**	0.03 (-0.01, 0.07)	**0.04 (0.003, 0.08)**	0.03 (-0.01, 0.07)	**0.07 (0.02, 0.13)**
Scale Portion						
Subscale	Intercept	Event	Symptom	Event x Symptom	Standard Slope	Deviant Slope
BPRS						
Activation	2.35 (2.27, 2.44)	0.01 (-0.01, 0.03)	**0.02 (0.002, 0.05)**	-0.003 (-0.01, 0.002)	**0.02 (0.002, 0.05)**	0.02 (-0.002, 0.04)
Affect	2.46 (2.40, 2.53)	**0.02 (0.001, 0.03)**	-0.003 (-0.01, 0.01)	**-0.002 (-0.004, -0.0002)**	-0.003 (-0.01, 0.01)	-0.01 (-0.01, 0.004)
Neg. Symptoms	2.4 (2.33, 2.47)	0.01 (-0.01, 0.03)	0.01 (-0.004 0.02)	-0.002 (-0.01, 0.001)	0.01 (-0.004 0.02)	0.01 (-0.01, 0.02)
Pos. Symptoms	2.41 (2.35, 2.47)	0.01 (-0.01, 0.02)	0.01 (-0.003, 0.01)	-0.001 (-0.003, 0.001)	0.01 (-0.003, 0.01)	0.01 (-0.007, 0.01)
Resistance	2.42 (2.36, 2.48)	0.01 (-0.004, 0.03)	0.01 (-0.01, 0.02)	-0.002 (-0.01, 0.001)	0.01 (-0.01, 0.02)	0.003 (-0.01, 0.02)
SAPS/SANS						
Inexpressivity	2.43 (2.39, 2.46)	0.002 (-0.01, 0.01)	0.004 (-0.00003, 0.01)	-0.0002 (0.001, 0.01)	0.004 (-0.00003, 0.01)	0.004 (-0.001, 0.01)
Reality Distortion	2.44 (2.41, 2.47)	0.004 (-0.004, 0.01)	0.004 (-0.004, 0.01)	-0.001 (-0.004, 0.01)	0.004 (-0.004, 0.01)	0.002 (-0.01, 0.01)
Apathy/Asociality	2.43 (2.38, 2.47)	0.004 (-0.01, 0.02)	0.002 (-0.002, 0.01)	-0.0004 (-0.001, 0.01)	0.002 (-0.002, 0.01)	0.002 (-0.002, 0.01)
Thought Disorder	2.42 (2.38, 2.46)	0.001 (-0.01, 0.01)	**0.01 (0.0004, 0.01)**	0.0003 (-0.001, 0.002)	**0.01 (0.0004, 0.01)**	**0.01 (0.0003, 0.01)**

Note: Instances where the 95% CrI does not contain zero are bolded for ease of interpretation.

Supplementary Table 14

Parameter Estimates and Simple Slopes for P3a-Symptom Models

Location Portion						
Subscale	Intercept	Event	Symptom	Event x Symptom	Target Slope	Novel Slope
BPRS						
Activation	0.63 (-0.57, 1.84)	**2.61 (1.52, 3.70)**	0.27 (-0.03, 0.57)	-0.2 (-0.48, 0.07)	0.27 (-0.03, 0.57)	0.07 (-0.26, 0.39)
Affect	0.79 (-0.14, 1.75)	**2.36 (1.49, 3.22)**	**0.12 (0.001, 0.23)**	-0.07 (-0.18, 0.04)	**0.12 (0.001, 0.23)**	0.05 (-0.08, 0.18)
Neg. Symptoms	1.8 (0.80, 2.79)	**2.17 (1.29, 3.07)**	-0.03 (-0.20, 0.14)	-0.06 (-0.21, 0.09)	-0.03 (-0.20, 0.14)	-0.09 (-0.28, 0.10)
Pos. Symptoms	1.14 (0.29, 1.97)	**1.93 (1.16, 2.69)**	0.09 (-0.04, 0.22)	-0.01 (-0.13, 0.11)	0.09 (-0.04, 0.22)	0.08 (-0.07, 0.22)
Resistance	1.05 (0.16, 1.93)	**2.18 (1.36, 2.98)**	0.13 (-0.04, 0.31)	-0.07 (-0.23, 0.09)	0.13 (-0.04, 0.31)	0.06 (-0.13, 0.26)
SAPS/SANS						
Inexpressivity	1.67 (1.17, 2.18)	**1.92 (1.46, 2.38)**	-0.01 (-0.07, 0.05)	-0.01 (-0.07, 0.04)	-0.01 (-0.07, 0.05)	-0.02 (-0.09, 0.05)
Reality Distortion	1.56 (1.11, 2.02)	**1.9 (1.48, 2.31)**	0.05 (-0.06, 0.16)	-0.03 (-0.12, 0.08)	0.05 (-0.06, 0.16)	0.02 (-0.10, 0.14)
Apathy/Asociality	1.52 (0.91, 2.14)	**2.05 (1.50, 2.62)**	0.01 (-0.04, 0.07)	-0.02 (-0.07, 0.02)	0.01 (-0.04, 0.07)	-0.01 (-0.07, 0.05)
Thought Disorder	1.51 (1.00, 2.02)	**1.82 (1.35, 2.30)**	0.04 (-0.05, 0.14)	0.01 (-0.07, 0.10)	0.04 (-0.05, 0.14)	0.05 (-0.05, 0.16)
Scale Portion						
Subscale	Intercept	Event	Symptom	Event x Symptom	Target Slope	Novel Slope
BPRS						
Activation	2.29 (2.20, 2.38)	0.08 (-0.01, 0.17)	0.01 (-0.01, 0.04)	**-0.02 (-0.05, -0.003)**	0.01 (-0.01, 0.04)	-0.01 (-0.03, 0.01)
Affect	2.33 (2.26, 2.40)	-0.03 (-0.10, 0.03)	0.002 (-0.01, 0.01)	0.003 (-0.01, 0.01)	0.002 (-0.01, 0.01)	0.005 (-0.004, 0.01)
Neg. Symptoms	2.36 (2.28, 2.44)	0.01 (-0.06, 0.09)	-0.003 (-0.02, 0.01)	-0.005 (-0.02, 0.01)	-0.003 (-0.02, 0.01)	-0.01 (-0.02, 0.01)
Pos. Symptoms	2.35 (2.28, 2.41)	-0.02 (-0.08, 0.04)	-0.0003 (-0.01, 0.01)	0.001 (-0.01, 0.01)	-0.0003 (-0.01, 0.01)	0.001 (-0.01, 0.01)
Resistance	2.37 (2.30, 2.44)	-0.03 (-0.09, 0.03)	-0.01 (-0.02, 0.01)	0.004 (-0.01, 0.02)	-0.01 (-0.02, 0.01)	-0.001 (-0.01, 0.01)
SAPS/SANS						
Inexpressivity	2.34 (2.30, 2.38)	-0.001 (-0.04, 0.03)	0.001 (-0.004, 0.01)	-0.002 (-0.01, 0.002)	0.001 (-0.004, 0.01)	-0.002 (-0.01, 0.003)
Reality Distortion	2.34 (2.31, 2.38)	-0.01 (-0.04, 0.02)	0.002 (-0.01, 0.01)	-0.001 (-0.01, 0.01)	0.002 (-0.01, 0.01)	0.002 (-0.01, 0.01)
Apathy/Asociality	2.34 (2.29, 2.39)	0.01 (-0.04, 0.05)	0.001 (-0.004, 0.005)	-0.002 (-0.01, 0.002)	0.001 (-0.004, 0.005)	-0.001 (-0.01, 0.003)
Thought Disorder	2.34 (2.30, 2.38)	-0.01 (-0.05, 0.02)	0.0001 (-0.01, 0.01)	0.001 (-0.01, 0.01)	0.0001 (-0.01, 0.01)	0.001 (-0.01, 0.01)

Note: Instances where the 95% CrI does not contain zero are bolded for ease of interpretation.

Supplementary Table 15

Parameter Estimates and Simple Slopes for P3a Collapsed Localizer-Symptom Models

Location Portion						
Subscale	Intercept	Event	Symptom	Event x Symptom	Target Slope	Novel Slope
BPRS						
Activation	2.83 (1.35, 4.32)	**4.22 (2.82, 5.64)**	**0.4 (0.03, 0.77)**	**-0.43 (-0.79, -0.08)**	**0.4 (0.03, 0.77)**	-0.03 (-0.42, 0.35)
Affect	3.17 (1.98, 4.38)	**3.72 (2.62, 4.82)**	**0.16 (0.01, 0.31)**	**-0.15 (-0.29, -0.01)**	**0.16 (0.01, 0.31)**	0.01 (-0.14, 0.16)
Neg. Symptoms	4.63 (3.37, 5.92)	**3.49 (2.30, 4.69)**	-0.06 (-0.28, 0.15)	-0.16 (-0.36, 0.04)	-0.06 (-0.28, 0.15)	-0.22 (-0.44, 0.001)
Pos. Symptoms	3.98 (2.90, 5.06)	**3.17 (2.17, 4.19)**	0.06 (-0.11, 0.23)	-0.1 (-0.26, 0.06)	0.06 (-0.11, 0.23)	-0.04 (-0.21, 0.14)
Resistance	3.78 (2.65, 4.92)	**3.37 (2.33, 4.42)**	0.12 (-0.11, 0.34)	-0.17 (-0.38, 0.04)	0.12 (-0.11, 0.34)	-0.05 (-0.28, 0.18)
SAPS/SANS						
Inexpressivity	4.41 (3.77, 5.06)	**2.83 (2.23, 3.43)**	-0.02 (-0.10, 0.06)	-0.05 (-0.12, 0.02)	-0.02 (-0.10, 0.06)	-0.07 (-0.15, 0.01)
Reality Distortion	4.28 (3.68, 4.87)	**2.78 (2.24, 3.33)**	0.02 (-0.12, 0.17)	-0.11 (-0.23, 0.03)	0.02 (-0.12, 0.17)	-0.08 (-0.22, 0.06)
Apathy/Asociality	4.38 (3.57, 5.20)	**3.09 (2.33, 3.83)**	-0.01 (-0.08, 0.06)	-0.05 (-0.12, 0.01)	-0.01 (-0.08, 0.06)	-0.06 (-0.13, 0.01)
Thought Disorder	4.21 (3.54, 4.87)	**2.65 (2.04, 3.29)**	0.03 (-0.09, 0.16)	-0.01 (-0.12, 0.10)	0.03 (-0.09, 0.16)	0.02 (-0.10, 0.14)
Scale Portion						
Subscale	Intercept	Event	Symptom	Event x Symptom	Target Slope	Novel Slope
BPRS						
Activation	2.46 (2.36, 2.55)	0.06 (-0.03, 0.15)	0.01 (-0.02, 0.03)	-0.02 (-0.04, 0.003)	0.01 (-0.02, 0.03)	-0.01 (-0.03, 0.01)
Affect	2.48 (2.40, 2.55)	-0.04 (-0.11, 0.02)	0.001 (-0.01, 0.01)	0.004 (-0.004, 0.01)	0.001 (-0.01, 0.01)	0.01 (-0.002, 0.01)
Neg. Symptoms	2.52 (2.44, 2.60)	-0.02 (-0.09, 0.06)	-0.01 (-0.02, 0.01)	0.001 (-0.01, 0.01)	-0.01 (-0.02, 0.01)	-0.01 (-0.02, 0.01)
Pos. Symptoms	2.5 (2.43, 2.56)	-0.02 (-0.08, 0.04)	-0.002 (-0.01, 0.01)	0.001 (-0.01, 0.01)	-0.002 (-0.01, 0.01)	-0.0003 (-0.01, 0.01)
Resistance	2.52 (2.45, 2.59)	-0.01 (-0.08, 0.05)	-0.01 (-0.02, 0.01)	0.0002 (-0.01, 0.01)	-0.01 (-0.02, 0.01)	-0.01 (-0.02, 0.01)
SAPS/SANS						
Inexpressivity	2.49 (2.45, 2.53)	-0.01 (-0.05, 0.03)	-0.0004 (-0.01, 0.004)	-0.001 (-0.01, 0.004)	-0.0004 (-0.01, 0.004)	-0.001 (-0.01, 0.003)
Reality Distortion	2.49 (2.45, 2.52)	-0.01 (-0.04, 0.02)	-0.0001 (-0.01, 0.01)	-0.0004 (-0.01, 0.01)	-0.0001 (-0.01, 0.01)	-0.001 (-0.01, 0.01)
Apathy/Asociality	2.49 (2.44, 2.54)	-0.003 (-0.05, 0.04)	0.0001 (-0.004, 0.004)	-0.001 (-0.004, 0.003)	0.0001 (-0.004, 0.004)	-0.001 (-0.01, 0.003)
Thought Disorder	2.49 (2.45, 2.53)	-0.01 (-0.05, 0.03)	0.0001 (-0.01, 0.01)	0.0001 (-0.01, 0.01)	0.0001 (-0.01, 0.01)	-0.0001 (-0.01, 0.01)

Note: Instances where the 95% CrI does not contain zero are bolded for ease of interpretation.

Supplementary Table 16

Parameter Estimates and Simple Slopes for P3b-Symptom Models

Location Portion						
Subscale	Intercept	Event	Symptom	Event x Symptom	Target Slope	Non-target Slope
BPRS						
Activation	1.26 (0.13, 2.38)	-0.13 (-1.15, 0.91)	**0.34 (0.06, 0.62)**	**-0.3 (-0.57, -0.05)**	**0.34 (0.06, 0.62)**	0.04 (-0.11, 0.18)
Affect	1.82 (0.95, 2.72)	-0.64 (-1.45, 0.14)	0.1 (-0.01, 0.20)	-0.08 (-0.18, 0.02)	0.1 (-0.01, 0.20)	0.01 (-0.04, 0.07)
Neg. Symptoms	2.87 (1.92, 3.82)	**-1.21 (-2.07, -0.35)**	-0.07 (-0.22, 0.09)	-0.01 (-0.15, 0.13)	-0.07 (-0.22, 0.09)	-0.07 (-0.16, 0.01)
Pos. Symptoms	2.22 (1.41, 3.01)	**-1.06 (-1.78, -0.34)**	0.05 (-0.07, 0.18)	-0.03 (-0.15, 0.08)	0.05 (-0.07, 0.18)	0.02 (-0.05, 0.09)
Resistance	2.14 (1.30, 2.99)	**-0.8 (-1.56, -0.05)**	0.09 (-0.08, 0.25)	-0.1 (-0.26, 0.05)	0.09 (-0.08, 0.25)	-0.02 (-0.11, 0.07)
SAPS/SANS						
Inexpressivity	2.63 (2.16, 3.10)	**-1.3 (-1.75, -0.88)**	-0.02 (-0.08, 0.03)	0.01 (-0.04, 0.06)	-0.02 (-0.08, 0.03)	-0.01 (-0.04, 0.02)
Reality Distortion	2.51 (2.08, 2.95)	**-1.24 (-1.63, -0.84)**	0.003 (-0.10, 0.11)	-0.01 (-0.11, 0.08)	0.003 (-0.10, 0.11)	-0.01 (-0.06, 0.05)
Apathy/Asociality	2.69 (2.10, 3.27)	**-1.34 (-1.89, -0.81)**	-0.02 (-0.07, 0.03)	0.01 (-0.04, 0.06)	-0.02 (-0.07, 0.03)	-0.01 (-0.03, 0.02)
Thought Disorder	2.4 (1.92, 2.90)	**-1.22 (-1.67, -0.77)**	0.04 (-0.05, 0.13)	-0.01 (-0.09, 0.07)	0.04 (-0.05, 0.13)	0.03 (-0.02, 0.07)
Scale Portion						
Subscale	Intercept	Event	Symptom	Event x Symptom	Target Slope	Non-target Slope
BPRS						
Activation	2.26 (2.16, 2.37)	-0.03 (-0.10, 0.05)	0.02 (-0.01, 0.04)	0.001 (-0.02, 0.02)	0.02 (-0.01, 0.04)	0.02 (-0.004, 0.04)
Affect	2.34 (2.26, 2.43)	-0.04 (-0.10, 0.02)	-0.002 (-0.01, 0.01)	0.002 (-0.01, 0.01)	-0.002 (-0.01, 0.01)	-0.00001 (-0.01, 0.01)
Neg. Symptoms	2.35 (2.26, 2.44)	0.03 (-0.04, 0.09)	-0.004 (-0.02, 0.01)	-0.01 (-0.02, 0.001)	-0.004 (-0.02, 0.01)	-0.01 (-0.03, 0.001)
Pos. Symptoms	2.33 (2.26, 2.41)	-0.04 (-0.09, 0.01)	-0.001 (-0.01, 0.01)	0.003 (-0.01, 0.01)	-0.001 (-0.01, 0.01)	0.002 (-0.01, 0.01)
Resistance	2.34 (2.26, 2.41)	**-0.05 (-0.11, -0.001)**	-0.002 (-0.02, 0.01)	0.01 (-0.004, 0.02)	-0.002 (-0.02, 0.01)	0.01 (-0.01, 0.02)
SAPS/SANS						
Inexpressivity	2.33 (2.29, 2.37)	-0.01 (-0.04, 0.02)	0.0001 (-0.01, 0.01)	-0.003 (-0.01, 0.001)	0.0001 (-0.01, 0.01)	-0.003 (-0.01, 0.002)
Reality Distortion	2.33 (2.29, 2.37)	-0.02 (-0.05, 0.002)	0.002 (-0.01, 0.01)	0.001 (-0.01, 0.01)	0.002 (-0.01, 0.01)	0.003 (-0.01, 0.01)
Apathy/Asociality	2.33 (2.28, 2.38)	-0.03 (-0.06, 0.01)	0.004 (-0.004, 0.004)	0.0004 (-0.003, 0.004)	0.004 (-0.004, 0.004)	0.0003 (-0.004, 0.01)
Thought Disorder	2.33 (2.29, 2.38)	**-0.03 (-0.07, -0.004)**	-0.002 (-0.01, 0.01)	0.004 (-0.002, 0.01)	-0.002 (-0.01, 0.01)	0.002 (-0.01, 0.01)

Note: Instances where the 95% CrI does not contain zero are bolded for ease of interpretation.

Supplementary Table 17

Parameter Estimates and Simple Slopes for P3b Collapsed Localizer-Symptom Models

Location Portion						
Subscale	Intercept	Event	Symptom	Event x Symptom	Target Slope	Non-target Slope
BPRS						
Activation	4.09 (2.72, 5.49)	**-3.06 (-4.37, -1.75)**	0.32 (-0.03, 0.68)	-0.28 (-0.62, 0.05)	0.32 (-0.03, 0.68)	0.04 (-0.11, 0.19)
Affect	4.57 (3.48, 5.67)	**-3.59 (-4.63, -2.55)**	0.1 (-0.03, 0.23)	-0.07 (-0.20, 0.06)	0.1 (-0.03, 0.23)	0.03 (-0.03, 0.09)
Neg. Symptoms	5.68 (4.48, 6.90)	**-4.05 (-5.17, -2.92)**	-0.07 (-0.27, 0.13)	-0.01 (-0.20, 0.17)	-0.07 (-0.27, 0.13)	-0.08 (-0.17, 0.0004)
Pos. Symptoms	5.24 (4.22, 6.25)	**-4.23 (-5.18, -3.27)**	0.01 (-0.15, 0.17)	0.02 (-0.13, 0.17)	0.01 (-0.15, 0.17)	0.03 (-0.04, 0.10)
Resistance	5.14 (4.08, 6.17)	**-3.92 (-4.90, -2.95)**	0.04 (-0.17, 0.25)	-0.05 (-0.24, 0.15)	0.04 (-0.17, 0.25)	-0.01 (-0.10, 0.08)
SAPS/SANS						
Inexpressivity	5.53 (4.92, 6.13)	**-4.26 (-4.82, -3.69)**	-0.05 (-0.13, 0.02)	0.03 (-0.04, 0.10)	-0.05 (-0.13, 0.02)	-0.02 (-0.05, 0.01)
Reality Distortion	5.35 (4.80, 5.91)	**-4.17 (-4.70, -3.65)**	-0.04 (-0.17, 0.10)	0.04 (-0.09, 0.16)	-0.04 (-0.17, 0.10)	0 (-0.06, 0.06)
Apathy/Asociality	5.72 (4.96, 6.48)	**-4.5 (-5.19, -3.79)**	-0.05 (-0.11, 0.01)	0.04 (-0.01, 0.10)	-0.05 (-0.11, 0.01)	-0.01 (-0.03, 0.02)
Thought Disorder	5.31 (4.69, 5.94)	**-4.22 (-4.80, -3.63)**	-0.01 (-0.12, 0.11)	0.03 (-0.08, 0.14)	-0.01 (-0.12, 0.11)	0.03 (-0.02, 0.07)
Scale Portion						
Subscale	Intercept	Event	Symptom	Event x Symptom	Target Slope	Non-target Slope
BPRS						
Activation	2.4 (2.30, 2.50)	**-0.08 (-0.15, -0.003)**	0.01 (-0.01, 0.04)	0.004 (-0.02, 0.02)	0.01 (-0.01, 0.04)	0.02 (-0.004, 0.04)
Affect	2.48 (2.40, 2.56)	**-0.09 (-0.14, -0.03)**	-0.003 (-0.01, 0.01)	0.003 (-0.004, 0.01)	-0.003 (-0.01, 0.01)	0.0002 (-0.01, 0.01)
Neg. Symptoms	2.46 (2.37, 2.55)	-0.01 (-0.07, 0.06)	-0.001 (-0.02, 0.01)	-0.01 (-0.02, 0.0002)	-0.001 (-0.02, 0.01)	-0.01 (-0.02, 0.002)
Pos. Symptoms	2.45 (2.38, 2.53)	**-0.08 (-0.13, -0.02)**	0.0001 (-0.01, 0.01)	0.002 (-0.01, 0.01)	0.0001 (-0.01, 0.01)	0.002 (-0.01, 0.01)
Resistance	2.45 (2.38, 2.53)	**-0.09 (-0.14, -0.03)**	0.0003 (-0.01, 0.02)	0.01 (-0.01, 0.02)	0.0003 (-0.01, 0.02)	0.01 (-0.01, 0.02)
SAPS/SANS						
Inexpressivity	2.45 (2.41, 2.49)	**-0.05 (-0.08, -0.02)**	0.001 (-0.004, 0.01)	-0.004 (-0.01, 0.0002)	0.001 (-0.004, 0.01)	-0.003 (-0.01, 0.002)
Reality Distortion	2.45 (2.41, 2.49)	**-0.06 (-0.09, -0.04)**	0.002 (-0.01, 0.01)	0.0006 (-0.01, 0.01)	0.002 (-0.01, 0.01)	0.003 (-0.01, 0.01)
Apathy/Asociality	2.45 (2.40, 2.50)	**-0.06 (-0.10, -0.02)**	0.0004 (-0.004, 0.01)	-0.0002 (-0.003, 0.003)	0.0004 (-0.004, 0.01)	0.0003 (-0.004, 0.004)
Thought Disorder	2.46 (2.41, 2.50)	**-0.07 (-0.10, -0.04)**	-0.0004 (-0.01, 0.01)	0.002 (-0.004, 0.01)	-0.0004 (-0.01, 0.01)	0.002 (-0.01, 0.01)

Note: Instances where the 95% CrI does not contain zero are bolded for ease of interpretation.

Supplementary Table 18

Parameter Estimates and Simple Slopes for ERN-Symptom Models

Location Portion						
Subscale	Intercept	Event	Symptom	Event x Symptom	Correct Slope	Error Slope
BPRS						
Activation	4.48 (2.45, 6.45)	**-4.06 (-5.72, -2.40)**	0.03 (-0.46, 0.54)	0.27 (-0.15, 0.68)	0.03 (-0.46, 0.54)	0.3 (-0.15, 0.76)
Affect	5.21 (3.63, 6.72)	**-3.66 (-4.97, -2.32)**	-0.09 (-0.27, 0.11)	0.09 (-0.07, 0.25)	-0.09 (-0.27, 0.11)	0.003 (-0.17, 0.18)
Neg. Symptoms	7.07 (5.36, 8.67)	**-4.57 (-5.93, -3.20)**	**-0.45 (-0.72, -0.17)**	**0.28 (0.06, 0.50)**	**-0.45 (-0.72, -0.17)**	-0.17 (-0.42, 0.08)
Pos. Symptoms	5.43 (4.02, 6.81)	**-3.92 (-5.08, -2.76)**	-0.15 (-0.36, 0.06)	0.16 (-0.01, 0.33)	-0.15 (-0.36, 0.06)	0.01 (-0.18, 0.19)
Resistance	6.3 (4.87, 7.82)	**-3.7 (-4.95, -2.43)**	**-0.39 (-0.67, -0.10)**	0.16 (-0.09, 0.39)	**-0.39 (-0.67, -0.10)**	-0.23 (-0.50, 0.03)
SAPS/SANS						
Inexpressivity	5.49 (4.63, 6.33)	**-3.77 (-4.49, -3.04)**	**-0.19 (-0.28, -0.09)**	**0.15 (0.07, 0.24)**	**-0.19 (-0.28, -0.09)**	-0.03 (-0.12, 0.06)
Reality Distortion	4.92 (4.16, 5.70)	**-3.26 (-3.91, -2.59)**	**-0.22 (-0.41, -0.03)**	0.15 (-0.001, 0.30)	**-0.22 (-0.41, -0.03)**	-0.07 (-0.23, 0.10)
Apathy/Asociality	6.07 (4.96, 7.17)	**-4.45 (-5.34, -3.57)**	**-0.17 (-0.27, -0.08)**	**0.16 (0.09, 0.24)**	**-0.17 (-0.27, -0.08)**	-0.01 (-0.09, 0.07)
Thought Disorder	5.22 (4.29, 6.16)	**-3.72 (-4.48, -2.96)**	**-0.19 (-0.34, -0.02)**	**0.2 (0.07, 0.33)**	**-0.19 (-0.34, -0.02)**	0.01 (-0.13, 0.15)
Scale Portion						
Subscale	Intercept	Event	Symptom	Event x Symptom	Correct Slope	Error Slope
BPRS						
Activation	2.36 (2.22, 2.51)	0.001 (-0.07, 0.07)	0.02 (-0.02, 0.05)	0.003 (-0.01, 0.02)	0.02 (-0.02, 0.05)	0.02 (-0.01, 0.05)
Affect	2.46 (2.34, 2.57)	0.01 (-0.04, 0.06)	-0.005 (-0.02, 0.01)	0.001 (-0.01, 0.01)	-0.005 (-0.02, 0.01)	-0.004 (-0.02, 0.01)
Neg. Symptoms	2.44 (2.31, 2.56)	0.01 (-0.04, 0.07)	-0.003 (-0.02, 0.02)	0.0003 (-0.01, 0.01)	-0.003 (-0.02, 0.02)	-0.003 (-0.02, 0.02)
Pos. Symptoms	2.42 (2.32, 2.52)	0.01 (-0.03, 0.06)	0.0001 (-0.01, 0.02)	-0.0001 (-0.01, 0.01)	0.0001 (-0.01, 0.02)	0.0001 (-0.01, 0.01)
Resistance	2.48 (2.38, 2.59)	0.02 (-0.03, 0.07)	-0.01 (-0.03, 0.01)	-0.002 (-0.01, 0.01)	-0.01 (-0.03, 0.01)	-0.02 (-0.03, 0.003)
SAPS/SANS						
Inexpressivity	2.42 (2.35, 2.48)	0.02 (-0.01, 0.04)	0.002 (-0.01, 0.01)	-0.001 (-0.004, 0.002)	0.002 (-0.01, 0.01)	0.001 (-0.01, 0.01)
Reality Distortion	2.43 (2.37, 2.48)	0.01 (-0.01, 0.04)	-0.002 (-0.01, 0.01)	-0.001 (-0.01, 0.004)	-0.002 (-0.01, 0.01)	-0.003 (-0.01, 0.01)
Apathy/Asociality	2.4 (2.32, 2.48)	0.03 (-0.01, 0.06)	0.003 (-0.003, 0.01)	-0.002 (-0.004, 0.001)	0.003 (-0.003, 0.01)	0.001 (-0.01, 0.01)
Thought Disorder	2.41 (2.35, 2.48)	0.02 (-0.01, 0.05)	0.005 (-0.01, 0.02)	-0.001 (-0.01, 0.004)	0.005 (-0.01, 0.02)	0.004 (-0.01, 0.01)

Note: Instances where the 95% CrI does not contain zero are bolded for ease of interpretation.

Supplementary Table 19

Parameter Estimates and Simple Slopes for RewP-Symptom Models

Location Portion						
Subscale	Intercept	Event	Symptom	Event x Symptom	Loss Slope	Gain Slope
BPRS						
Activation	6.46 (4.34, 8.62)	**2.85 (1.26, 4.44)**	0.55 (-0.01, 1.10)	-0.05 (-0.46, 0.37)	0.55 (-0.01, 1.10)	0.5 (-0.15, 1.17)
Affect	6.84 (5.27, 8.42)	**2.96 (1.83, 4.09)**	**0.23 (0.03, 0.43)**	-0.04 (-0.18, 0.10)	**0.23 (0.03, 0.43)**	0.19 (-0.04, 0.43)
Neg. Symptoms	7.82 (6.00, 9.61)	**2.6 (1.32, 3.87)**	0.12 (-0.18, 0.43)	0.02 (-0.20, 0.23)	0.12 (-0.18, 0.43)	0.14 (-0.22, 0.50)
Pos. Symptoms	8.11 (6.63, 9.55)	**2.17 (1.14, 3.21)**	0.07 (-0.17, 0.31)	0.09 (-0.07, 0.26)	0.07 (-0.17, 0.31)	0.16 (-0.12, 0.44)
Resistance	8.71 (7.20, 10.21)	**1.9 (0.84, 2.99)**	-0.06 (-0.36, 0.24)	0.18 (-0.04, 0.39)	-0.06 (-0.36, 0.24)	0.12 (-0.24, 0.48)
SAPS/SANS						
Inexpressivity	8.25 (7.35, 9.10)	**2.81 (2.19, 3.43)**	0.05 (-0.05, 0.15)	-0.03 (-0.10, 0.04)	0.05 (-0.05, 0.15)	0.02 (-0.10, 0.14)
Reality Distortion	8.54 (7.74, 9.34)	**2.59 (2.01, 3.15)**	-0.05 (-0.25, 0.16)	0.07 (-0.07, 0.21)	-0.05 (-0.25, 0.16)	0.02 (-0.22, 0.27)
Apathy/Asociality	8.08 (7.02, 9.15)	**3.07 (2.31, 3.83)**	0.05 (-0.05, 0.14)	-0.05 (-0.11, 0.02)	0.05 (-0.05, 0.14)	0 (-0.11, 0.11)
Thought Disorder	8.08 (7.19, 8.96)	**2.64 (2.00, 3.28)**	0.13 (-0.04, 0.30)	0.01 (-0.11, 0.14)	0.13 (-0.04, 0.30)	0.14 (-0.06, 0.34)
Scale Portion						
Subscale	Intercept	Event	Symptom	Event x Symptom	Loss Slope	Gain Slope
BPRS						
Activation	2.24 (2.12, 2.36)	0.02 (-0.08, 0.12)	0.02 (-0.01, 0.05)	0.002 (-0.02, 0.03)	0.02 (-0.01, 0.05)	0.02 (-0.01, 0.05)
Affect	2.28 (2.19, 2.36)	0.02 (-0.05, 0.09)	0.01 (-0.01, 0.02)	0.0004 (-0.01, 0.01)	0.01 (-0.01, 0.02)	0.01 (-0.00, 0.02)
Neg. Symptoms	2.32 (2.22, 2.42)	0.01 (-0.07, 0.10)	-0.001 (-0.02, 0.02)	0.002 (-0.01, 0.02)	-0.001 (-0.02, 0.02)	0.002 (-0.01, 0.02)
Pos. Symptoms	2.29 (2.21, 2.36)	0.04 (-0.03, 0.11)	0.01 (-0.01, 0.02)	-0.003 (-0.01, 0.01)	0.01 (-0.01, 0.02)	0.003 (-0.01, 0.02)
Resistance	2.31 (2.23, 2.38)	0.03 (-0.04, 0.09)	0.003 (-0.01, 0.02)	-0.001 (-0.01, 0.01)	0.003 (-0.01, 0.02)	0.002 (-0.01, 0.02)
SAPS/SANS						
Inexpressivity	2.33 (2.28, 2.37)	0.02 (-0.01, 0.06)	-0.002 (-0.01, 0.004)	0.00001 (-0.004, 0.004)	-0.002 (-0.01, 0.004)	-0.002 (-0.01, 0.003)
Reality Distortion	2.32 (2.28, 2.36)	0.02 (-0.01, 0.06)	0.0004 (-0.01, 0.01)	0.0002 (-0.01, 0.01)	0.0004 (-0.01, 0.01)	0.001 (-0.01, 0.01)
Apathy/Asociality	2.33 (2.27, 2.38)	0.03 (-0.02, 0.08)	-0.001 (-0.01, 0.004)	-0.0003 (-0.004, 0.004)	-0.001 (-0.01, 0.004)	-0.001 (-0.01, 0.003)
Thought Disorder	2.29 (2.24, 2.34)	0.03 (-0.01, 0.07)	**0.01 (0.0001, 0.02)**	-0.003 (-0.01, 0.005)	**0.01 (0.0001, 0.02)**	0.01 (-0.002, 0.01)

Note: Instances where the 95% CrI does not contain zero are bolded for ease of interpretation.

Supplementary Table 20

Post-Error Slowing Parameter and Simple Slope Contrasts

ERN Simple Slopes				
	Location		Scale	
Patient	Median	95% CrI	Median	95% CrI
ERN-Congruent	-0.34	(-0.94, 0.26)	-0.002	(-0.01, 0.002)
ERN-Incongruent	-0.25	(-0.85, 0.27)	0.0001	(-0.002, 0.002)
ERN-Difference	0.09	(-0.75, 0.89)	0.002	(-0.001, 0.01)
Control				
ERN-Congruent	0.19	(-0.14, 0.53)	-0.001	(-0.004, 0.003)
ERN-Incongruent	-0.17	(-0.58, 0.30)	0.004	(0.0003, 0.01)
ERN-Difference	0.36	(-0.19, 0.88)	-0.004	(-0.01, 0.001)
**ERN Simple Slope Contrasts**				
	Location		Scale	
	Median	95% CrI	Median	95% CrI
Congruent: Patient-Control	-0.53	(-1.22, 0.15)	-0.002	(-0.01, 0.003)
Incongruent: Patient-Control	-0.09	(-0.83, 0.58)	-0.003	(-0.01, 0.001)
Difference: Patient-Control	-0.28	(-1.26, 0.71)	**0.01**	**(0.0004, 0.01)**
**Parameter Contrasts**				
	Location		Scale	
	Median	95% CrI	Median	95% CrI
Congruent: Patient-Control	**49.39**	**(19.43, 78.54)**	**0.24**	**(0.09, 0.37)**
Incongruent: Patient-Control	**47.48**	**(14.61, 79.21)**	**0.27**	**(0.14, 0.40)**
Difference: Patient-Control	1.85	(-15.01, 19.10)	-0.04	(-0.19, 0.11)

Note: ERN simple slopes represent the relationship between previous-trial ERN and reaction time (RT) for congruent and incongruent trials and their difference (incongruent minus congruent). All contrasts are for the patient minus control contrasts. If the 95% CrI of the contrast excludes zero, this is interpreted as evidence of a difference. In such instances, the contrast is shown in bold font.

Supplementary Table 21

Pairwise Contrasts for Group-Related Differences for Patients with Schizophrenia versus Healthy Controls

Location Portion of Models					
	MMN-D			MMN-F	
Parameter	Median	95% CrI	Parameter	Median	95% CrI
Standard	0.16	(-0.36, 0.68)	Standard	**0.68**	**(0.13, 1.25)**
Deviant	**0.92**	**(0.19, 1.66)**	Deviant	**1.36**	**(0.55, 2.18)**
Deviant-Standard	**0.76**	**(0.21, 1.31)**	Deviant-Standard	**0.68**	**(0.09, 1.26)**
	P3a			P3b	
Parameter	Median	95% CrI	Parameter	Median	95% CrI
Target	0.58	(-0.57, 1.68)	Target	-0.18	(-1.28, 0.93)
Novel	0.58	(-0.61, 1.78)	Nontarget	-0.47	(-1.10, 0.14)
Novel-Target	0.001	(-1.00, 0.99)	Target-Nontarget	*-0.3*	(-1.31, 0.72)
	ERN			RewP	
Parameter	Median	95% CrI	Parameter	Median	95% CrI
Correct	**-4.44**	**(-6.57, -2.30)**	Loss	0.96	(-1.07, 3.09)
Error	-1.58	(-3.63, 0.49)	Gain	0.86	(-1.48, 3.21)
Error-Correct	**2.87**	**(1.17, 4.52)**	Loss-Gain	-0.11	(-1.57, 1.35)
Scale Portion of Models					
	MMN-D			MMN-F	
Parameter	Median	95% CrI	Parameter	Median	95% CrI
Standard	**0.11**	**(0.01, 0.22)**	Standard	0.1	(-0.01, 0.20)
Deviant	0.1	(-0.01, 0.21)	Deviant	**0.11**	**(0.01, 0.21)**
Deviant-Standard	-0.01	(-0.03, 0.01)	Deviant-Standard	0.01	(-0.01, 0.03)
	P3a			P3b	
Parameter	Median	95% CrI	Parameter	Median	95% CrI
Target	0.01	(-0.09, 0.11)	Target	0.03	(-0.07, 0.13)
Novel	0.0003	(-0.10, 0.10)	Nontarget	0.02	(-0.07, 0.11)
Novel-Target	-0.01	(-0.09, 0.08)	Target-Nontarget	-0.01	(-0.08, 0.06)
	RewP			ERN	
Parameter	Median	95% CrI	Parameter	Median	95% CrI
Loss	-0.01	(-0.12, 0.10)	Correct	0.06	(-0.11, 0.23)
Gain	-0.01	(-0.13, 0.10)	Error	0.04	(-0.11, 0.19)
Loss-Gain	-0.01	(-0.10, 0.09)	Error-Correct	-0.02	(-0.11, 0.06)

Note: All estimates are for the patient minus control contrasts. If the 95% CrI of the contrast excludes zero, this is interpreted as evidence of a difference. In such instances, the contrast is shown in bold font. RewP = Reward Positivity; ERN = Error-related Negativity; MMN-D = Mismatch Negativity Duration; MMN-F: Mismatch Negativity Frequency

Supplementary Table 22

Estimates from Location-Scale Multilevel Model Predicting MMN-D Amplitude Including Only Patients with Schizophrenia and Healthy Controls

Predictor	Estimate	*SE*	95% CrI
Location Portion			
Standard: Controls (Intercept)	1.72	0.14	1.44, 2.00
Standard: Patients	0.16	0.26	-0.36, 068
Deviant: Controls	-2.62	0.15	-2.92, -2.33
Deviant: Patients	0.76	0.28	0.21, 1.31
Scale Portion (*SD*)			
Standard: Controls (Intercept)	2.49	0.03	2.44, 2.55
Standard: Patients	0.11	0.05	0.01, 0.22
Deviant: Controls	0.01	0.01	-0.001, 0.02
Deviant: Patients	-0.01	0.01	-0.03, 0.01
Random Effects (*SD*)			
Mean Structure			
Standard (Intercept)	1.54	0.11	1.34, 1.77
Deviant	1.40	0.10	1.21, 1.62
Variance Structure			
Standard (Intercept)	0.32	0.02	0.29, 0.35
Deviant	0.03	0.01	0.01, 0.04

Note: Estimates of parameters represent the median, and parameters in standard deviations (*SD*) units are shown on a log scale. *SE* = standard error; 95% CrI = 95% credible interval

Supplementary Table 23

Estimates from Location-Scale Multilevel Model Predicting MMN-F Amplitude Including Only Patients with Schizophrenia and Healthy Controls

Predictor	Estimate	*SE*	95% CrI
Location Portion			
Standard: Controls (Intercept)	-0.41	0.15	-0.70, -0.11
Standard: Patients	0.69	0.28	0.13, 1.25
Deviant: Controls	-3.42	0.16	-3.73, -3.11
Deviant: Patients	0.67	0.30	0.09, 1.26
Scale Portion (*SD*)			
Standard: Controls (Intercept)	2.43	0.03	2.37, 2.48
Standard: Patients	0.11	0.05	0.01, 0.21
Deviant: Controls	0.01	0.01	-0.004, 0.02
Deviant: Patients	-0.01	0.01	-0.03, 0.01
Random Effects (*SD*)			
Mean Structure			
Standard (Intercept)	1.73	0.11	1.53, 1.95
Deviant	1.56	0.11	1.36, 1.78
Variance Structure			
Standard (Intercept)	0.30	0.02	0.27, 0.34
Deviant	0.04	0.01	0.03, 0.06

Note: Estimates of parameters represent the median, and parameters in standard deviations (*SD*) units are shown on a log scale. *SE* = standard error; 95% CrI = 95% credible interval

Figure Captions

Supplementary Figure 1. Data retention for participants based on the following inclusion criteria: 1) complete diagnostic information verified by clinical interview, 2) clinical symptom ratings with less than 10% missing data, 3) at least five trials for each ERP condition. Additional participants were excluded from analyses due to unreadable EEG files.

Supplementary Figure 2. Grand average stimulus-locked waveforms at Fz for mismatch negativity-duration deviant (MMN-D; Panels A & B) and at Fz for mismatch negativity-frequency deviant (MMN-F; Panels C & D). Difference waveforms represent duration deviant minus standard activity for MMN-D (B) and frequency deviant minus standard for MMN-F (D). Shaded regions represent time-windows used for scoring.

Supplementary Figure 3. Grand average stimulus-locked waveforms at Cz for P3a (Panels A & B) and at Pz for P3b (Panels C & D). Difference waveforms represent novel minus target activity for P3a (B) and nontarget minus target activity for P3b (D). Shaded regions represent time-windows used for scoring. These temporal windows did not capture P3a and P3b optimally, and a collapsed localizer approach was used to identify alternative windows: 385 to 485 ms for P3a and 425 to 525 ms for P3b. The pattern of effects was similar for statistical analyses of scores using these alternative windows (see supplementary material).

Supplementary Figure 4. Grand average stimulus-locked waveforms at FCz for reward positivity (RewP) during the doors task (Panels A & B), and grand average response-locked waveforms at FCz for error-related negativity (ERN) during the flanker task (Panels C & D). Difference

waveforms represent gain minus loss activity for the doors task (B) and error minus correct activity for the flanker task (D). Shaded regions represent time-windows used for scoring.

References

1. Shafer A (2005): Meta-analysis of the brief psychiatric rating scale factor structure. *Psychological Assessment*. 17:324-335.
2. Kotov R, Foti D, Li K, Bromet EJ, Hajcak G, Ruggero CJ (2016): Validating dimensions of psychosis symptomatology: Neural correlates and 20-year outcomes. *Journal of Abnormal Psychology*. 125:1103-1119.
3. Longenecker JM, Haas GL, Salisbury DF (2022): Hierarchical symptom components in early psychosis. *Schizophrenia Bulletin*. 48:893-901.
4. Donaldson KR, Larsen EM, Jonas K, Tramazzo S, Perlman G, Foti D, et al. (2021): Mismatch negativity amplitude in first-degree relatives of individuals with psychotic disorders: Links with cognition and schizotypy. *Schizophrenia Research*. 238:161-169.
5. Eriksen BA, Eriksen CW (1974): Effects of noise letters upon the identification of a target letter in a non-search task. *Perception & Psychophysics*. 16:143-149.
6. Foti D, Kotov R, Bromet E, Hajcak G (2012): Beyond the broken error-related negativity: Functional and diagnostic correlates of error processing in psychosis. *Biological Psychiatry*. 71:864-872.
7. Perlman G, Foti D, Jackson F, Kotov R, Constantino E, Hajcak G (2015): Clinical significance of auditory target P300 subcomponents in psychosis: Differential diagnosis, symptom profiles, and course. *Schizophrenia Research*. 165:145-151.
8. Lopez-Calderon J, Luck SJ (2014): ERPLAB: An open-source toolbox for the analysis of event-related potentials. *Frontiers in Human Neuroscience*. 8:213.
9. Dien J (2010): The ERP PCA Toolkit: An open source program for advanced statistical analysis of event-related potential data. *Journal of Neuroscience Methods*. 187:138-145.
10. Delorme A, Makeig S (2004): EEGLAB: An open source toolbox for analysis of single-trial EEG dynamics including independent component analsis. *Journal of Neuroscience Methods*. 134:9-21.
11. Perrin F, Pernier J, Bertrand O, Echallier JF (1989): Spherical splines for scalp potential and current-density mapping. *Electroencephalography and Clinical Neurophysiology*. 72:184-187.
12. Luck SJ, Gaspelin N (2017): How to get statistically significant effects in any ERP experiment (and why you shouldn't). *Psychophysiology*. 54:146-157.
13. Wilkinson GN, Rogers CE (1973): Symbolic description of factorial models for analysis of variance. *Journal of the Royal Statistical Society Series C (Applied Statistics)*. 22:392-399.
14. R Development Core Team (2021): R: A language and environment for statistical computing. *R Foundation for Statistical Computing*. Vienna, Austria.
15. Bürkner P-C (2017): brms: An R package for Bayesian multilevel models using Stan. *Journal of Statistical Software*. 80:1-28.
16. Stan Development Team (2021): Stan Modeling Language Users Guide and Reference Manual v2.26.1.
17. Lewandowski D, Kurowicka D, Joe H (2009): Generating random correlation matrices based on vines and extended onion method. *Journal of Multivariate Analysis*. 100:1989-2001.
18. Clayson PE, Rocha HA, Baldwin SA, Rast P, Larson MJ (2022): Understanding the error in psychopathology: Notable intraindividual differences in neural variability of performance monitoring. *Biological Psychiatry: Cognitive Neuroscience and Neuroimaging*. 7:555-565.
